# Nogo-A regulates myogenesis via interacting with Filamin-C

**DOI:** 10.1038/s41420-020-00384-x

**Published:** 2021-01-06

**Authors:** SunYoung Park, Ji-Hwan Park, Un-Beom Kang, Seong-Kyoon Choi, Ahmed Elfadl, H. M. Arif Ullah, Myung-Jin Chung, Ji-Yoon Son, Hyun Ho Yun, Jae-Min Park, Jae-hyuk Yim, Seung-Jun Jung, Sang-Hyup Kim, Young-Chul Choi, Dae-Seong Kim, Jin-Hong Shin, Jin-Sung Park, Keun Hur, Sang-Han Lee, Eun-Joo Lee, Daehee Hwang, Kyu-Shik Jeong

**Affiliations:** 1 Department of Pathology, College of Veterinary Medicine, Kyungpook National University, Daegu, 41566 Republic of Korea; 2 Stem Cell Therapeutic Research Institute, Kyungpook National University, Daegu, 41566 Republic of Korea; 3 Department of New Biology, DGIST, Daegu, 42988 Republic of Korea; 4 R&D Division, BERTIS, Inc., Seongnam-si, Gyeonggi-do 13605 Republic of Korea; 5 Division of Biotechnology, DGIST, Daegu, 42988 Republic of Korea; 6 Core Protein Resources Center, DGIST, Daegu, 42988 Republic of Korea; 7 Department of Neurology, Gangnam Severance Hospital, Yonsei University College of Medicine, Seoul, 06058 Republic of Korea; 8 Department of Neurology, Pusan National University Yangsan Hospital, Yangsan, 50612 Republic of Korea; 9 Department of Neurology, Kyungpook National University School of Medicine, Daegu, 41944 Republic of Korea; 10 Department of Biochemistry and Cell Biology, Kyungpook National University School of Medicine, Daegu, 41944 Republic of Korea; 11 Department of Food Science and Biotechnology, Kyungpook National University, Daegu, 41566 Republic of Korea; 12 Department of Biological Sciences, Seoul National University, Seoul, 08826 Republic of Korea

**Keywords:** Cytoskeleton, Cell signalling

## Abstract

Among the three isoforms encoded by *Rtn4*, Nogo-A has been intensely investigated as a central nervous system inhibitor. Although Nogo-A expression is increased in muscles of patients with amyotrophic lateral sclerosis, its role in muscle homeostasis and regeneration is not well elucidated. In this study, we discovered a significant increase in Nogo-A expression in various muscle-related pathological conditions. Nogo^−/−^ mice displayed dystrophic muscle structure, dysregulated muscle regeneration following injury, and altered gene expression involving lipid storage and muscle cell differentiation. We hypothesized that increased Nogo-A levels might regulate muscle regeneration. Differentiating myoblasts exhibited Nogo-A upregulation and silencing Nogo-A abrogated myoblast differentiation. Nogo-A interacted with filamin-C, suggesting a role for Nogo-A in cytoskeletal arrangement during myogenesis. In conclusion, Nogo-A maintains muscle homeostasis and integrity, and pathologically altered Nogo-A expression mediates muscle regeneration, suggesting Nogo-A as a novel target for the treatment of myopathies in clinical settings.

## Introduction

Muscular dystrophies are a group of genetic muscle disorders that cause defective muscle fiber function due to the weakening and breakdown of muscle fibers as a result of inflammation accompanied with abrogated muscle regeneration. Duchenne muscular dystrophy (DMD), the most common form of muscular dystrophy, is severe myopathy associated with an abrogated dystrophin gene, which results in muscle membrane frailty associated with increased pro-inflammatory cytokine levels, mitochondrial dysfunction, and impaired satellite cell polarity^1–4^.

Following injury, satellite cells transform to committed myoblasts and ultimately differentiate into myofibers during myogenesis, a multistep process including transformation of satellite cells into myoblasts, differentiation into fusion-competent myoblasts, and their fusion into multinucleated myotubes^5,6^. Myogenesis is regulated by the synchronized actions of the myogenic regulatory factors myogenic factor 5, MyoD, and myogenin, and results in the expression of muscle-specific genes^7,8^. During differentiation, the Pax3 and Pax7 expression levels are downregulated, although satellite cells retain Pax7 expression during quiescence^9–11^.

The neurite outgrowth inhibitor Nogo, also known as reticulon 4, is encoded by *Rtn4*. Independently cloned by three groups^12–14^, Rtn4 complementary DNA (cDNA) was found to generate three Nogo isoforms, Nogo-A, Nogo-B, and Nogo-C, by alternative splicing or distinctive promoter usage^15,16^. Although mainly localized to the endoplasmic reticulum (ER), Nogo has also been observed on the cell surface^17^. Nogo-A, the largest Nogo isoform, which also carries an inhibitor region named Δ20, is a potent neurite growth inhibitor in the central nervous system (CNS)^14,15^. Nogo-A is expressed in the brain, spinal cord, eye, and skeletal muscle^18^, and most of our knowledge on Nogo-A concerns its function as a neurite outgrowth inhibitor in the CNS^13,19^. On the surface of cells, Nogo-A acts via its receptors Nogo-66 receptor 1, sphingosine-1-phosphate receptor (S1PR) 2, and myelin-associated glycoprotein. Nogo isoforms binds Nogo-66 receptor 1 and inhibits neurite growth via the Rho/Rho-associated kinase signaling pathway^14,18,20,21^. However, the mechanisms underlying the role of Nogo-A in muscle regeneration and myogenesis in skeletal muscle remain elusive.

In the present study, we also elucidate the role of Nogo in the regulation of muscle homeostasis, muscle differentiation, and adipocyte differentiation based on the muscle transcriptome profiles of Nogo^+/+^ and Nogo^−/−^ mice. We use a muscle-injury model in Nogo^−/−^ mice to examine Nogo-A involvement in muscle regeneration and further demonstrate the potent myogenic function of Nogo-A in myoblast differentiation using muscle lineage cells, including C2C12 cells and induced muscle stem cells (iMSCs). Our findings suggest that the expression of Nogo-A is altered in the skeletal muscle during pathological processes, and that Nogo-A serves a critical function in muscle regeneration and maintenance of muscle integrity.

## Results

### Nogo expression is altered in the muscle tissue of patients with myopathies

**Fig. 1:**
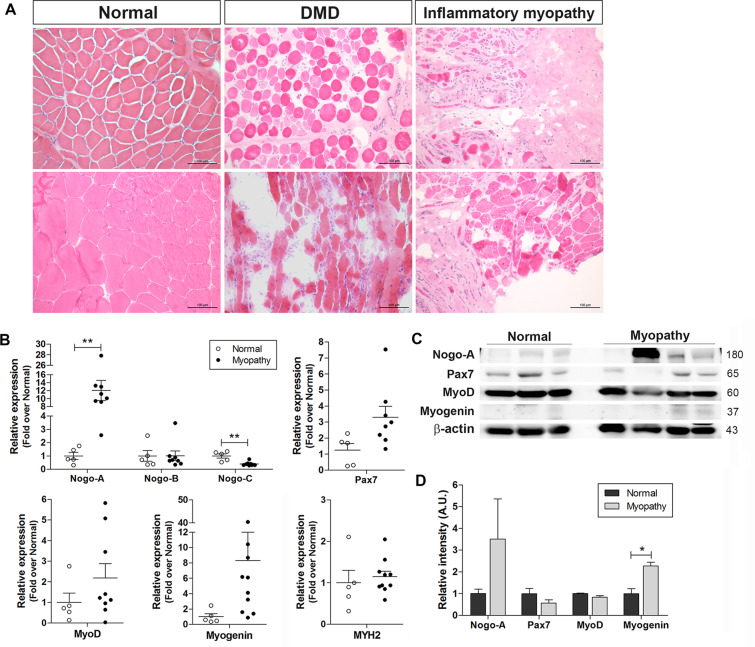
Altered expression of Nogo and myogenic factors in muscles under pathological or abnormal physiological states. **A** Hematoxylin/eosin staining of muscle biopsy samples from patients with Duchenne muscular dystrophy (DMD) and inflammatory myopathies and normal subjects. Scale bar = 100 µm. **B** Muscle mRNA samples from normal human subjects (*n* = 5) and patients with myopathy (*n* = 8–10) were evaluated by quantitative reverse-transcription PCR (qRT-PCR). Mean ± SEM. **p < 0.01. **C** Western blot (WB) analysis of indicated proteins in muscles tissue samples from normal subjects (*n* = 3) and myopathic patients (*n* = 4). Molecular weights (kDa) are indicated. **D** Quantitative assessment of band intensity (**C**) using the NIH ImageJ software. Mean ± SEM. *p < 0.05.

Muscle tissue samples from patients with different myopathies, including DMD and inflammatory myopathy, exhibited typical histopathological features of muscle degeneration including damaged myofiber structure, inflammatory cell infiltration, and fibrosis resulting from incomplete muscle regeneration (Fig. 1A). Thus, we examined the transcriptional expression levels of Nogo isoforms and myogenic factors in muscle tissues from patients with myopathies including DMD and inflammatory myopathy, which revealed that the levels of Nogo isoforms were significantly altered in the muscle samples of patients with myopathies (Fig. 1B). Specifically, although the level of Nogo-B in myopathic tissues was comparable to that in normal muscle tissues, the level of Nogo-A was dramatically elevated and the level of Nogo-C was significantly reduced in the myopathic tissues compared with the normal tissues (Fig. 1B). The transcript levels of Pax7, a satellite cell marker, and myogenic factors including MyoD and myogenin were upregulated in the myopathic tissues compared to the normal tissues (Fig. 1B), and the increased Nogo-A and myogenin transcript levels were associated with an elevation in the protein levels of Nogo-A and myogenin in the myopathic tissues (Fig. 1C, D).

### The expression levels of Nogo and myogenic factors are altered in animal models of muscle disorders

**Fig. 2:**
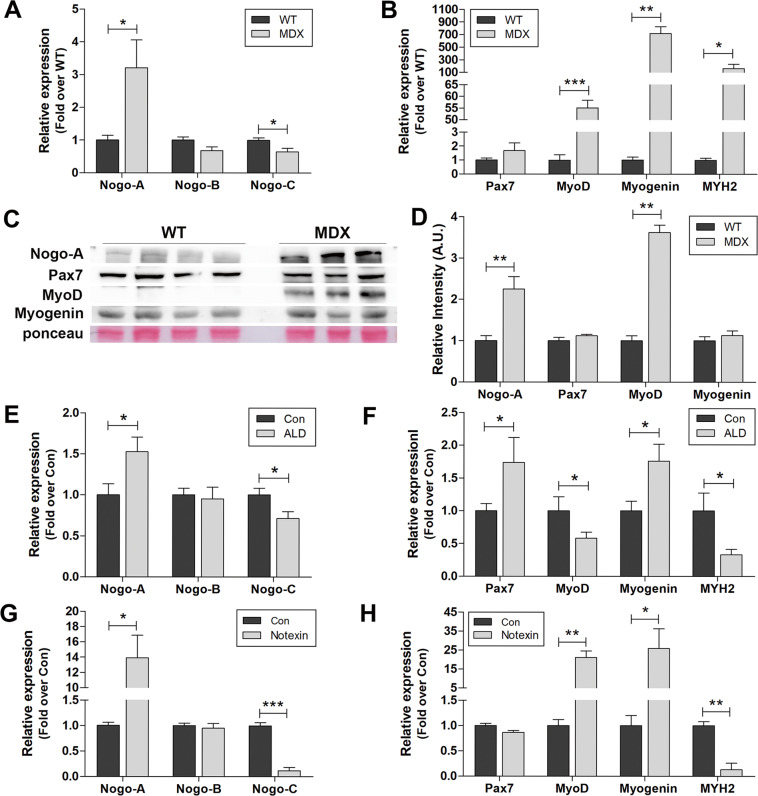
Altered expression of Nogo and myogenic factors in animal myopathy models. **A**, **B** qRT-PCR analysis for indicated genes in muscle samples from 12-week-old wild-type (WT) (*n* = 4) and *mdx* (*n* = 3) mice. Mean ± SEM. *p < 0.05, **p < 0.01, ***p < 0.001. **C** WB analysis of indicated proteins in muscle extracts from 12-week-old WT (*n* = 4) and *mdx* (*n* = 3) mice. Molecular weights (kDa) are indicated. **D** Quantitative assessment for protein (**C**) using the NIH ImageJ software. Mean ± SEM. **p < 0.01. **E**, **F** qRT-PCR analysis for indicated genes in muscle samples from control (Con, *n* = 6) mice in a model of alcoholic liver disease (ALD) (*n* = 8). Mean ± SEM. *p < 0.05. **G**, **H** qRT-PCR analysis for indicated genes in muscle samples from control (Con, *n* = 3) and notexin-injured mice (8-week-old C57BL/6, 50 ng/gastrocnemius, 3 days post injury, *n* = 3). Mean ± SEM. *p < 0.05, **p < 0.01, ***p < 0.001.

The muscles of *mdx* mice, the animal model of human DMD, showed significantly elevated mRNA levels of Nogo-A (Fig. 2A), MyoD, and myogenin (Fig. 2B), and a reduced Nogo-C mRNA level (Fig. 2A) compared with the wild-type (WT) mice. These results were consistent with our analyses in human muscle samples from myopathies (Fig. 1B). The observed upregulation in Nogo-A and Myod transcript levels was accompanied by increases in their protein levels (Fig. 2C, D). Dasarathy et al.^22^ have demonstrated a relationship between skeletal muscle loss and alcoholic liver disease (ALD); thus, we also analyzed the skeletal muscle in a mouse model of ALD as a myopathy model associated with chronic liver dysfunction. In current study, the mouse ALD model successfully exhibited liver pathology including hepatomegaly, determined by the ratio of liver weight to body weight (Fig. S1A), and elevated levels of plasma aspartate aminotransferase (AST) and alanine aminotransferase (ALT) (Fig. S1C) compared to the control mice. Therefore, the elevated plasma ALT and AST levels in ALD mice suggest liver dysfunction as well as muscle damage (Fig. S1C). The skeletal muscle tissues of the ALD mice exhibited upregulated Nogo-A and reduced Nogo-C mRNA levels compared to the controls (Fig. 2E); the tissues also showed elevated levels of myogenin and Pax7 (Fig. 2F), suggesting increased numbers of committed Pax7^+^ satellite cells and myogenin-positive myoblasts.

Acute muscle injury induced by notexin injection was associated with a dramatic increase in Nogo-A and a significant reduction in Nogo-C (Fig. 2G), as well as significant upregulation of Myod and myogenin (Fig. 2H). In these animals, the number of Pax7^+^ committed satellite cells was not altered but the MYH2 expression was dramatically downregulated, indicating structural muscle damage (Fig. 2H).

### Muscle lacking Nogo exhibits abnormal structure and impaired regeneration following notexin-induced muscle injury

**Fig. 3:**
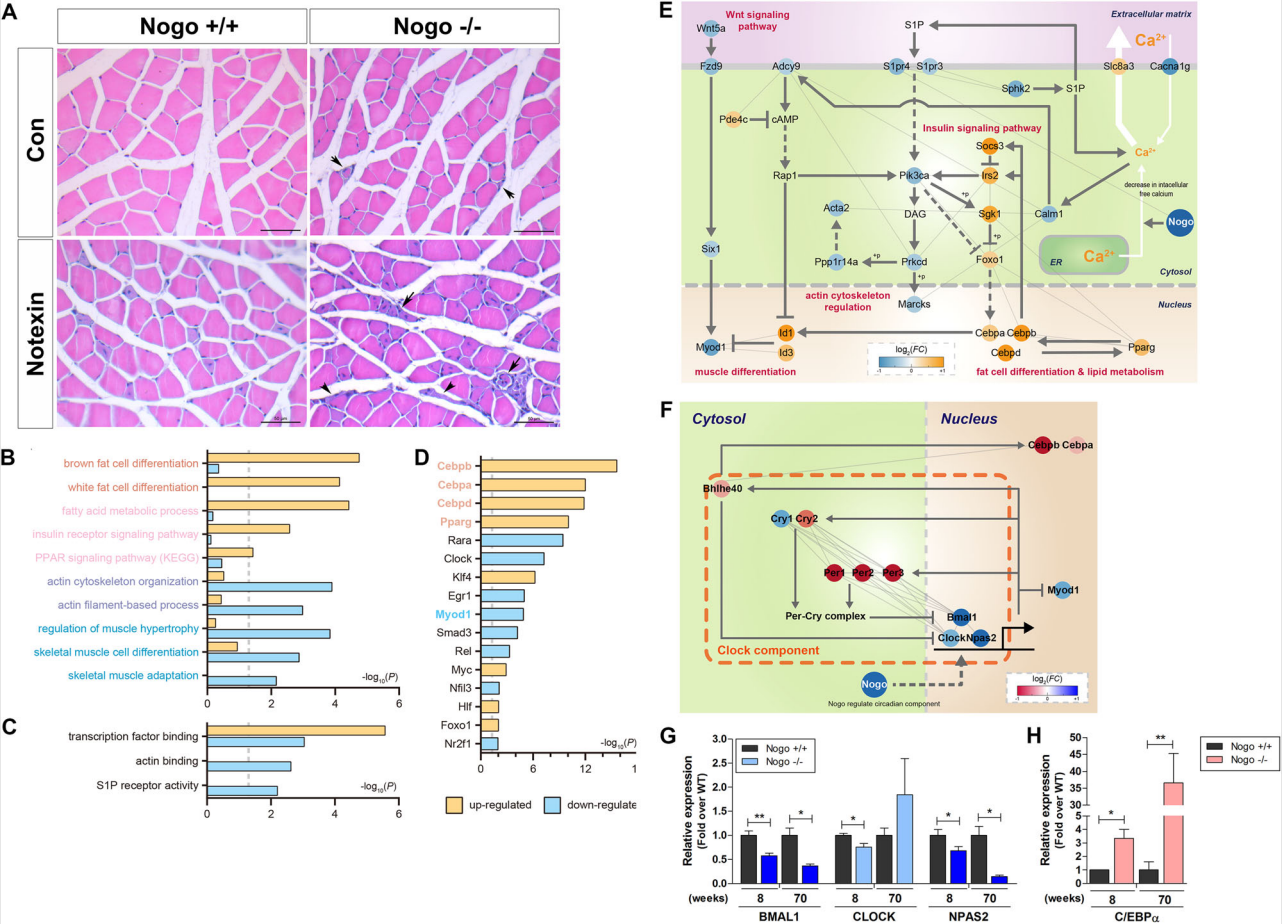
Cellular processes affected by Nogo deficiency, and muscle physiology of notexin-induced Nogo^−/−^ mice. **A** Representative images of hematoxylin/eosin stained non-injured control (Con, *n* = 5) and notexin-injured (500 ng/gastrocnemius, *n* = 5) muscles from Nogo^+/+^ or Nogo^−/−^ mice. Short and long arrows indicate defective and regenerating myofibers, respectively. Arrowheads mark fibrotic cells. Scale bar = 50 µm. Gene Ontology biological processes (GOBPs) (**B**) and gene Ontology molecular functions (GOMFs) (**C**) represented by genes up- and downregulated by Nogo knockout. Enrichment significance was determined via *p*-values calculated with DAVID is displayed as −log_10_(*P*). *P*-values for up- and downregulated GOBPs or GOMFs are denoted using orange or light blue bars, respectively. *p* < 0.05. **D** Major transcription factors (TFs) that regulate a high number of differentially regulated genes (DEGs). Four upregulated TFs and one downregulated TF in Nogo^−/−^ mice are labeled in orange and light blue, respectively. **E** Model network describing potential roles of Nogo in the regulation of myogenesis. Node colors represent upregulation (orange) and downregulation (blue) of the corresponding genes by the Nogo knockout. Arrows and suppression symbols denote activation and inhibition, respectively, based on the KEGG pathway database. Solid and dotted lines represent direct and indirect reactions, respectively, and gray lines represent protein–protein interactions between the connected nodes. The color bar represents gradient of log2 fold changes, log2(FC), between the Nogo^−/−^ and WT muscle tissues. **F** Network model for circadian rhythm regulation in Nogo^−/−^ mouse. Node colors represent upregulation (red) and downregulation (blue) of the corresponding genes by Nogo knockout. Arrows and suppression symbols denote activation and inhibition, respectively, based on the KEGG pathway database. Solid and dotted lines represent direct and indirect reactions, respectively, and gray lines represent protein–protein interactions between the connected nodes. The color bar represents gradient of log2(FC) between the Nogo^−/−^ and WT muscle tissues. **G**, **H** qRT-PCR analysis for indicated genes in muscle samples from 8- and 70-week-old Nogo^+/+^ (*n* = 4) and Nogo^−/−^ (*n* = 3) mice. Mean ± SEM. *p < 0.05, **p < 0.01.

Although the Nogo-A^+/+^ muscles exhibited well-structured muscle fibers and the absence of inflammatory cell infiltration, the Nogo^−/−^ muscles displayed defective myofibers and exhibited immune cell infiltration (Fig. 3A, right panel, arrow). Notexin-induced muscle damage results in inflammation at the site of injection 3 days post injury, with the completion of regeneration 28 days post injury^23^. The notexin-injured muscles from Nogo-A^+/+^ mice exhibited centrally nucleated fibers indicating regenerating myofibers and increased infiltration of inflammatory cells between the fibers at 8 days post injury (Fig. 3A, left). In contrast, the muscles from notexin-injured Nogo^−/−^ mice displayed a higher number of regenerated myofibers and inflammatory cells, suggesting the susceptibility of Nogo-deficient muscles to injury (Fig. 3A, right). In addition, myofibers with defective regeneration (Fig. 3A, right, arrow) and increased fibrotic cells (Fig. 3A, right, arrowhead) in injured Nogo^−/−^ muscles represented the atrophy phenotype.

### Absence of Nogo abrogates muscle differentiation and enhances fat cell differentiation and lipid metabolism-related gene expression

Comparison of the mRNA expression profiles between the two groups identified 703 differentially expressed genes (DEGs), including 355 genes that were upregulated and 348 genes that were downregulated in the Nogo^−/−^ muscles compared to the Nogo-A^+/+^ muscles (Table S1). To investigate potential cellular processes affected by Nogo, we conducted an enrichment analysis of Gene Ontology biological processes (GOBPs) and Gene Ontology molecular functions (GOMFs) using the DAVID software (Table S2). The upregulated genes in Nogo^−/−^ muscles were mainly involved in fat cell development; specifically, brown and white fat cell differentiation and lipid metabolism via the fatty acid metabolic process, the insulin receptor signaling pathway, and the peroxisome proliferator-activated receptor signaling pathway (Fig. 3B, C). The downregulated genes were mainly involved in cytoskeletal organization and included actin filament-base processing, actin cytoskeleton organization, actin binding, and muscle development via regulation of muscle hypertrophy, skeletal muscle adaptation, and cell differentiation (Fig. 3B). The GOMFs enriched by the downregulated genes included S1PR activity, which can induce myogenesis^24^ (Fig. 3C).

The TF enrichment analysis to uncover potential TFs regulated by Nogo (Fig. 3D and Table S3) revealed 17 major TFs regulating several DEGs. Among these, Nogo knockout (KO) upregulated four TFs known to regulate fat cell development, *Cebpa*, *Cebpb*, *Cebpd*, and *Pparg*^25,26^, but downregulated *Myod1*, a master TF for muscle development^27^ (Fig. 3D), suggesting that these TFs act downstream from Nogo and contribute to the attenuation of myotube development.

Finally, we built a network model describing the genes associated with the identified GOBPs and major TFs as indicated above to understand the functions of the DEGs and the five major TFs in attenuation of myotube development, which showed that Nogo KO downregulated Wnt signaling, indicated by the downregulation of *Wnt5a*, *Fzd9*, and *Six1*, resulting in decreased activation of *Myod1*^28^ (Fig. 3E, left). Consistent with this finding, negative regulators of *Myod1*, including *Id1/3*, and the upstream TFs *Cebpa*, *Cebpb*, *Cebpd*, and *Pparg* were upregulated by Nogo KO (Fig. 3E, bottom). The network model also indicated that Nogo KO altered the mRNA expression levels of *Slc8a3*, *Cacna1g*, and *Sphk2*, which are involved in decreasing the amount of cytosolic calcium (Fig. 3E, right). Consistent with this finding, Nogo KO further downregulated *Calm1* and *Adcy9a* in the downstream calcium signaling pathway, and *S1pr3/4*, *Pik3ca*, and *Prkcd* in the downstream sphingosine-1 phosphate phosphatidylinositol 3-kinase pathway (Fig. 3E, middle), resulting in decreased activation of cytoskeleton reorganization observed by downregulation of *Marcks*, *Ppp1r14a*, and *Acta2*. These data suggest that Nogo positively regulates Wnt, calcium, MyoD1, and S1P phosphatidylinositol 3-kinase signaling pathways, and negatively regulates insulin signaling, *Cebpa*, *Cebpb*, *Cebpd*, and *Pparg*, to coordinate myogenesis regulation.

Interestingly, genes related to circadian rhythm were significantly represented in both the up- and downregulated gene groups in the *Nogo*^*−/−*^ muscles (Fig. 3F). To elucidate the potential circadian clock activity resulting from Nogo KO, we mapped the identified DEGs to the Kyoto Encyclopedia of Genes and Genomes (KEGG) pathway database and identified the core clock components *Cry1*, *Cry2*, *Per1*, *Per2*, *Per3*, *Bhlhe40*, *Bmal1*, *Npas2*, and *Clock* as DEGs in the circadian rhythm pathway. Specifically, the CLOCK/BMAL1 inhibitors *Cry2*, *Per1*, *Per2*, *Per3*, and *Bhlhe40* were upregulated and the CLOCK/BMAL1 components *Bmal1*, *Npas2*, and *Clock* were downregulated. These clock components are known to regulate myogenesis^29–32^. The CLOCK/BMAL1 elements *Bmal1*, *Npas2*, and *Clock* bind to the *Myod1* enhancer to induce *Myod1* expression (Fig. 3E, left)^29,30^. *Cry1* and *Cry2* play opposing roles in myogenic differentiation: *Cry1* knockdown promotes myoblast differentiation, which is inhibited by *Cry2* knockdown (Fig. 3E, middle)^32^. In contrast, *Bhlhe40* regulates adipogenic differentiation by interacting with *Cebpb* (Fig. 3F, top)^31^. These models suggest that Nogo-A regulates myogenesis through the control of circadian clock gene expression. Further, muscles from 70-week-old mice were analyzed to delineate Nogo activity in circadian regulation and muscular fat cell deposition. As seen in Figs. 3G and 3H, the downregulation of *Bmal1* and *Npas2* was enhanced in aged *Nogo*^*−/−*^ muscles and was accompanied by an increase in *Cebpa*, suggesting that Nogo modulates fat deposition in muscle via circadian clock regulation.

### Nogo-A is highly expressed during late differentiation in C2C12 cells and is related to the expression of myogenic factors

**Fig. 4:**
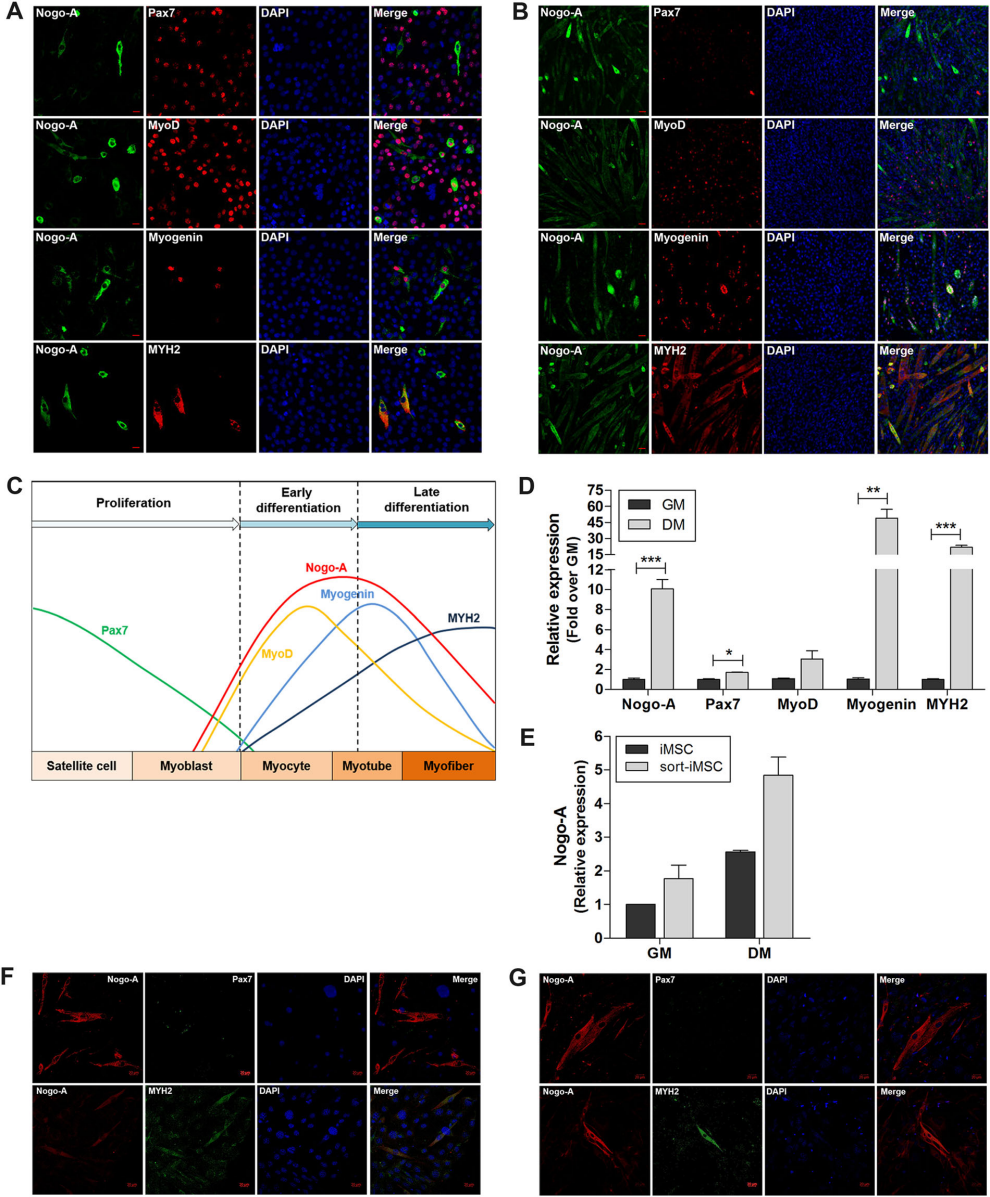
Enhanced expression of Nogo-A and myogenic factors during myoblast differentiation. Cells were cultured in growth medium (GM) to maintain a proliferation/early differentiation state; differentiation was induced by incubating cells with differentiation medium (DM) for 3 days. **A**, **B** IF staining of Nogo-A (green) with Pax7, MyoD, myogenin, or MYH2 (red) in C2C12 mouse myoblast cells in early differentiation (**A**) and late differentiation (**B**). Scale bar = 20 µm. **C** Summary of the expression levels of Nogo-A and myogenic factors during myoblast differentiation. **D** qRT-PCR analysis of Nogo-A and myogenic factors in myoblasts (*n* = 3/group). Mean ± SEM. *p < 0.05, **p < 0.01, ***p < 0.001. **E** qRT-PCR analysis of Nogo-A in induced muscle stem cells (iMSCs) and sorted-iMSCs (sort-iMSCs). **F**, **G** IF staining of Nogo-A (red) with Pax7 or MYH2 (green) in differentiated iMSCs (**F**) and sort-iMSCs (**G**). Scale bar = 20 µm.

Based on our observation that Nogo-A levels were increased under pathological conditions, we assessed the role of Nogo-A during myogenesis in C2C12 mouse myoblasts. During early differentiation, the expression levels of Pax7, a quiescent satellite cell marker, and MyoD, an early myogenic differentiation marker, were significant and Nogo-A expression was observed in Pax7^−^ and MyoD^+^ cells (Fig. 4A). The late differentiation marker myogenin as well as MYH2 were also observed in Nogo-A^+^ cells (Fig. 4B). Significant features of differentiated cells included a dramatic reduction in Pax7 and increases in MYH2 and Nogo-A (Fig. 4B). Analysis of the expression levels of Nogo-A and myogenic factors were well-matched to these observations (Fig. 4D). During differentiation, Nogo-A expression and its cytoplasmic localization corresponded to those of MYH2 (Fig. 4A, B). Previously, we have derived iMSCs from mouse embryo fibroblasts bearing potent myogenic differentiation capacity to show that iMSCs sorted according to stem cell markers (sort-iMSCs) had enhanced myogenic potential^33^. To confirm the expression of Nogo-A during myogenic differentiation, we analyzed differentiated iMSCs and sort-iMSCs, and observed enhanced *Nogo-A* expression (Fig. 4E) and Nogo-A colocalization with MYH2 during differentiation in both populations (Fig. 4F, G).

### Predicted TF binding to Nogo-A TF-binding sites is altered during differentiation in C2C12 cells

**Fig. 5:**
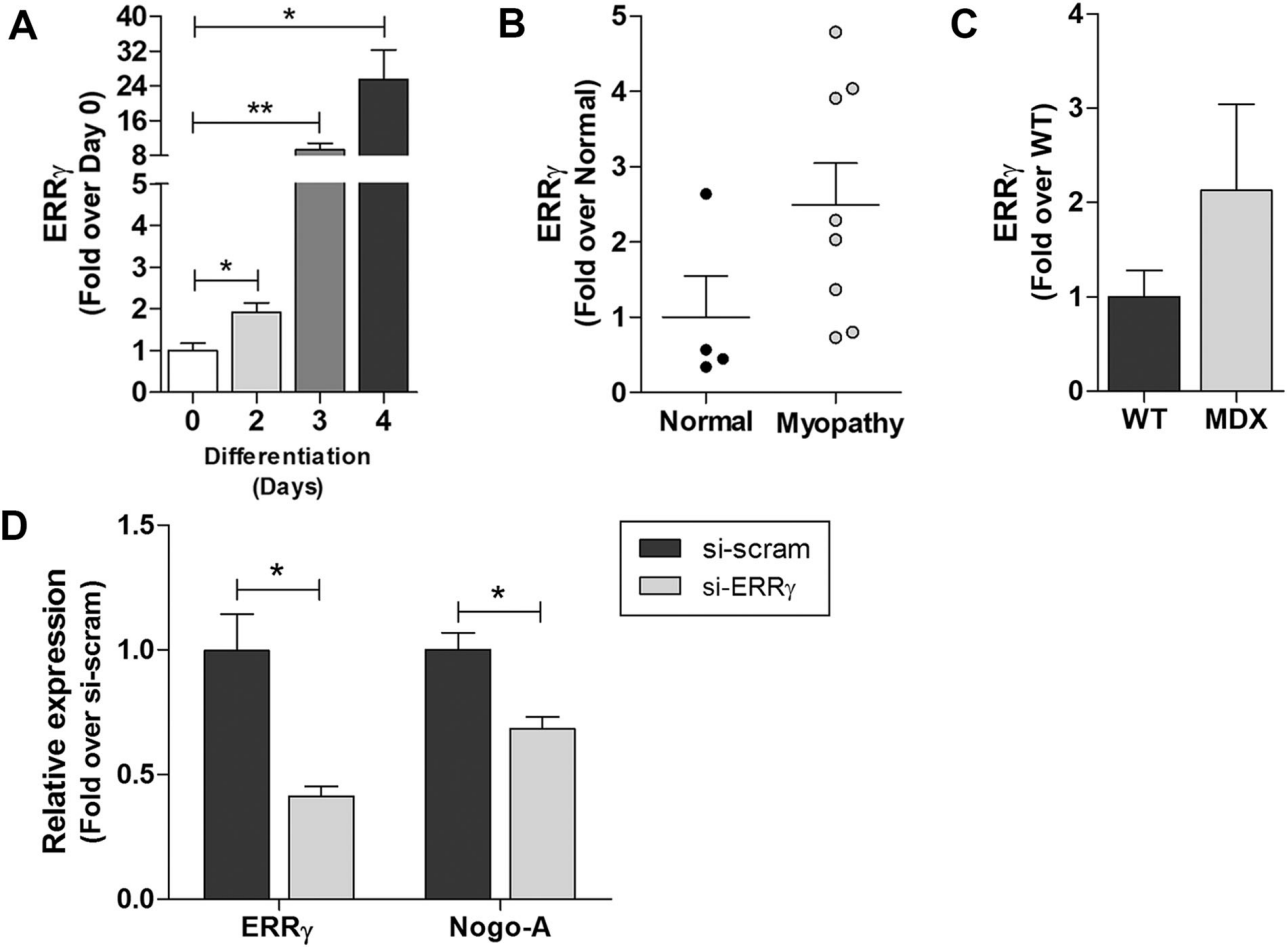
Expression of ERRγ in differentiated C2C12 cells and dystrophic muscles, and Nogo-A expression in ERRγ-silenced C2C12 cells. **A**–**C** qRT-PCR analysis of ERRγ during differentiation of C2C12 cells with DM (**A**), in normal (*n* = 4) and myopathic (*n* = 8) human muscle tissues (**B**), and of muscles from WT (*n* = 4) and *mdx* (*n* = 4) mice (**C**). **D** qRT-PCR analysis of si-scrambled- or si-ERRγ-transfected C2C12 cells (*n* = 3/group). Mean ± SEM. *p < 0.05.

We analyzed the Nogo TF-binding site to generate a list of TFs predicted to bind to the site (Table S4) and utilized differentiated C2C12 cells to assess the participation of predicted TFs in myoblast differentiation. Among the predicted TFs, the expression levels of *Errg*, *Arid3b*, *Arnt*, *Mlxip*, *Klf1*, and *Mycn* were significantly increased (Fig. 5A and Fig. S2). Further, the expression of ERRγ, a proposed critical regulator of myogenesis^33^, was elevated in human myopathic muscles (Fig. 5B, C). Then, we assessed the roles of these factors in Nogo-A transcription using small interfering RNAs (siRNAs) targeting *Errg* in C2C12 cells. As shown in Fig. 5D, the abrogated expression levels of ERRγ resulted in the reduction of Nogo-A, which suggests that an increase in Nogo-A expression during myogenesis may be regulated by the myogenic signals via ERRγ.

### Nogo-A functions in myogenesis

**Fig. 6:**
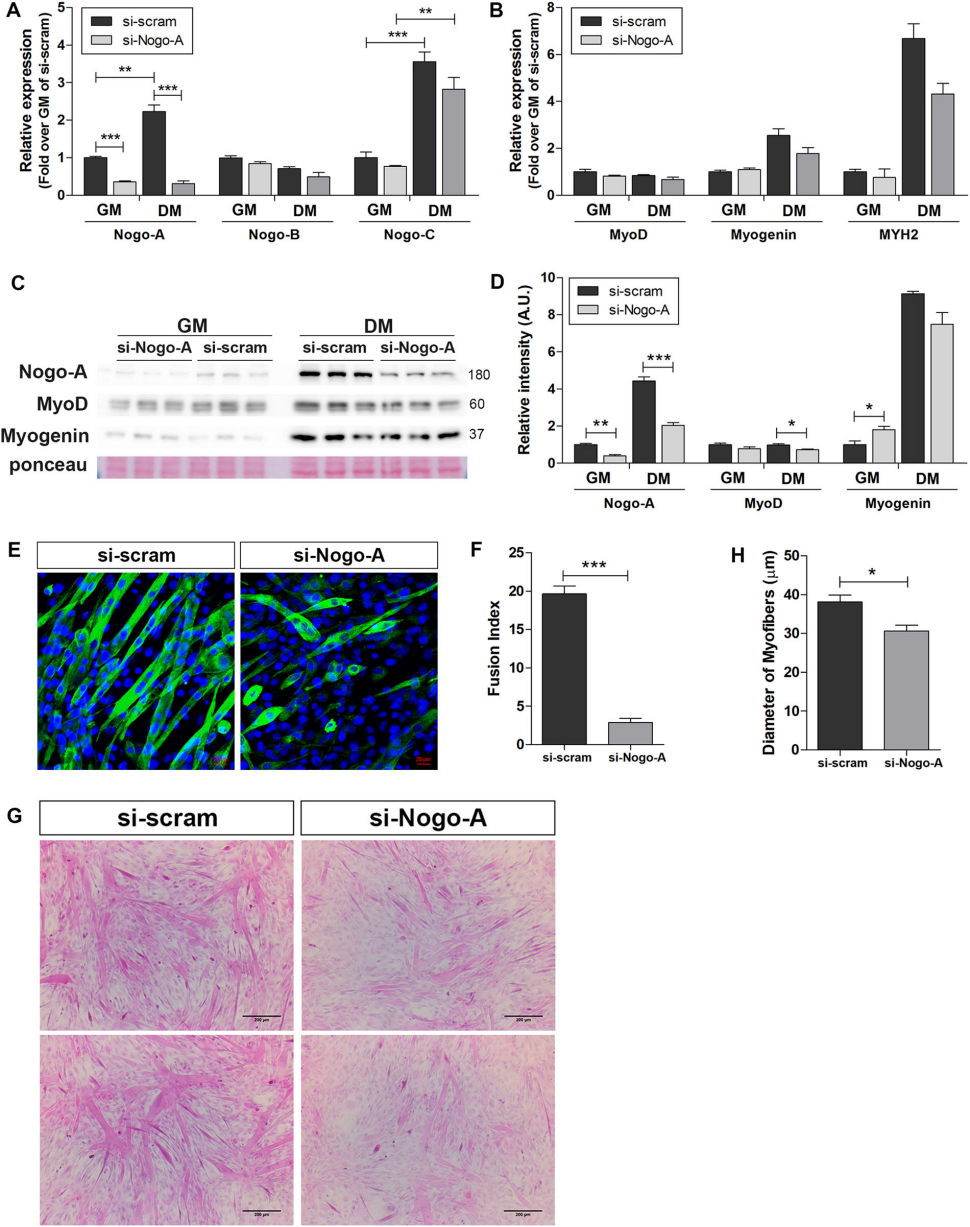
Defective myotube formation in Nogo-A-silenced C2C12 cells. Expression of Nogo-A in C2C12 myoblast was silenced using si-Nogo-A, and myoblast differentiation was induced by DM for 3 days. **A**, **B** qRT-PCR analysis of *Nogo* isoforms (**A**) and *Myod*, myogenin, and *Myh2* (**B**) in si-scrambled- or si-Nogo-A-transfected C2C12 cells (*n* = 3/group). Mean ± SEM. *p < 0.05, **p < 0.01, ***p < 0.001. **C** WB of indicated proteins in Nogo-A-silenced cells (*n* = 3/group). Molecular weights (kDa) are indicated. **D** Quantitative assessment of band intensities of WBs in **C** using the NIH ImageJ software. Mean ± SEM. **p < 0.01, ***p < 0.001. **E** IF staining of Nogo-A (green) with DAPI (blue) in differentiated si-scrambled- or si-Nogo-A-transfected C2C12 cells. Scale bar = 20 µm. **F** Fusion index of differentiated si-scrambled- or si-Nogo-A-transfected C2C12 cells. Fusion index was calculated as the percentage of total nuclei incorporated in myotubes. **G** Hematoxylin/eosin staining of differentiated si-scrambled- or si-Nogo-A-transfected C2C12 cells. Scale bar = 200 µm. **H** Quantitative assessment of myofiber diameter based on evaluation of 20 diameters in five fields per group) in L. Mean ± SEM. *p < 0.05.

To elucidate the role of Nogo-A during muscle differentiation, Nogo-A expression in C2C12 was abrogated by transfecting an siRNA targeting Nogo-A (si-Nogo-A), followed by differentiation induction to evaluate Nogo-A function during myogenesis. The Nogo-A expression level was significantly abrogated by si-Nogo-A (growth medium (GM), Fig. 6A) and was maintained during differentiation medium (DM, Fig. 6A). The effect of Nogo-A silencing by si-Nogo-A on the levels of Nogo-B, Nogo-C, MyoD, myogenin, and MYH2 was not significant (Fig. 6A, B). Silencing of Nogo-A significantly decreased the protein levels of Nogo-A and slightly downregulatedthe MyoD and myogenin during differentiation (Fig. 6C, D). Myotube development was attenuated by Nogo-A silencing (Fig. 6E), and the decreased myotube fusion index (Fig. 6F) and smaller-diameter myofibers (Fig. 6G, H) indicated immature myofiber formation in the Nogo-A-silenced cells. These results implicate the role of increased Nogo-A in myogenesis.

### Nogo-A localizes to cytoskeleton during myogenesis

**Fig. 7:**
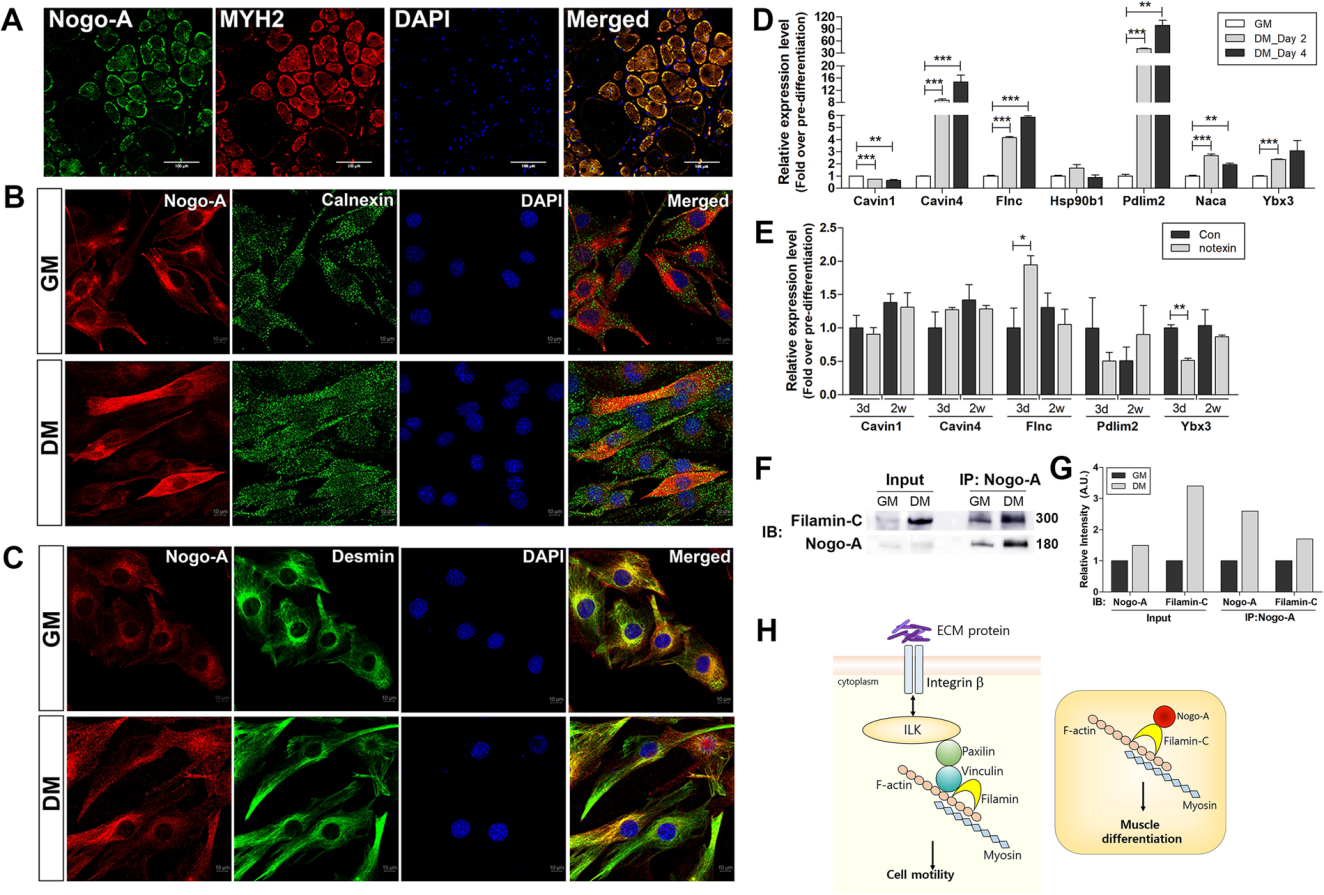
Location of Nogo-A in mature skeletal muscle and C2C12 myoblasts, and identification of Nogo-A interacting factors during differentiation. **A**–**C** Localization of Nogo-A in mature skeletal muscle and C2C12 myoblasts. **A** Isolated gastrocnemius muscles from 8-week-old C27BL/6 mouse were sectioned and stained for Nogo-A (green) and MYH2 (red). Scale bar = 100 µm. **B** IF staining of Nogo-A (red) and calnexin (ER protein, green) in growth-conditioned C2C12 cells. Scale bar = 10 µm. **C** IF staining of Nogo-A (red) and desmin (muscle-specific, type III intermediate filament, green) in differentiation-conditioned C2C12 cells. Scale bar = 10 µm. **D**–**F** Expression levels of Nogo-A-interacting molecules suggested by immunoprecipitation-mass spectrometry analysis of differentiated C2C12. qRT-PCR analysis of indicated genes in C2C12 cells cultured with DM for 2 or 4 days (**D**) and in muscles after 3 days or 2 weeks of notexin injury (**E**). **F** Immunoprecipitation with a Nogo-A-specific antibody was performed using lysates of C2C12 cells maintained in GM or DM for 4 days. WB analysis of indicated proteins in immunoprecipitated samples. Molecular weights (kDa) are indicated. **G** Quantitative assessment of band intensities of blots in **F** using the NIH ImageJ software. **H** Filamin-mediated cell motility regulation and proposed model of Nogo-A’s role in muscle differentiation via its interaction with filamin-C.

As shown in Fig. 7A, mature muscles exhibited robust expression of MYH2 and Nogo-A, which were colocalized. Nogo-A belongs to the reticulon family and mainly resides in ER; Nogo-A has a known inhibitory role in neurite growth when it is expressed on cell membrane via binding its receptors on oligodendrocytes^14,20,21^. Interestingly, Nogo-A was not colocalized with calnexin, implying that ER is not a prominent location for Nogo-A during the growth and differentiation stages of C2C12 cells (Fig. 7B). Desmin, a muscle-specific intermediate filament, staining was significant around the nucleus during growth; however, differentiated C2C12 cells exhibited stretched cytoskeletal location of desmin which was colocalized with Nogo-A (Fig. 7C). Thus, Nogo-A might play a role during myogenesis by interacting with the cytoskeletal network.

### Nogo-A activity in myogenesis occurs independently of its interaction with its cognate receptor

Nogo-A activity in the plasma membrane is induced via binding to the Nogo receptor NgR at the Nogo-66 region and S1PR2 at the Nogo-A-specific Δ20 region^34^. NgR is a common receptor bound by all three Nogo isoforms, whereas S1PR2 is specific to Nogo-A^35^. Thus, we explored the interaction between Nogo-A and S1PR2 in differentiated C2C12 cells to evaluate whether Nogo participates in myoblast differentiation in a membrane receptor-dependent manner. The expression of S1PR2 was not altered during differentiation and its interaction with Nogo-A was not observed in differentiated myoblasts (Fig. S3), indicating that Nogo-A functions in an S1PR2-independent manner in myoblast differentiation.

### Nogo-A interacts with several proteins involved in cell motility and cytoskeletal organization during myogenesis

Proteins common to both the cell lysates and samples immunoprecipitated with the Nogo-A-specific antibody were identified, and nonspecific proteins bound to the immunoglobulin G antibody were excluded to specify Nogo-A-interacting proteins. Finally, a list of interacting molecules categorized according to the differentiation state of cells was obtained by comparing Nogo-A-specific proteins between C2C12 cells in DM and those in GM (Table S5). The subcellular location of the listed molecules varied and included extracellular matrix, plasma membrane, cytoskeleton, cytoplasm, ER, mitochondrion, and nucleus. Analysis of the canonical pathways involving the Nogo-A-binding proteins revealed a significant correlation between signaling pathways regulating cell motility and cytoskeletal organization and the Nogo-A binding proteins (Table S6 and Fig. S4).

To elucidate the role of Nogo-A-binding proteins in myogenesis, we selected several molecules, including *Cavin1*, *Cavin4*, *Flnc*, *Pdlim2*, *Hsp90b1*, *Naca*, and *Ybx3*, which locate to the ER or cytoskeleton. During C2C12 differentiation, the expression levels of *Cavin4*, *Flnc*, *Pdlim2*, and *Ybx3* were increased whereas that of *Cavin1* was reduced (Fig. 7D). The levels of upregulated molecules during myoblast differentiation were evaluated in notexin-injured muscles, showing that *Flnc* was increased at 3 days post injury (Fig. 7E).

Finally, we evaluated the interaction between Nogo-A and filamin-C, the product of *Flnc*, during myogenesis by Nogo-A-specific antibody-mediated immunoprecipitation. C2C12 cells in DM showed significantly elevated levels of filamin-C and Nogo-A, and the interaction between the two proteins was enhanced compared to that in C2C12 cells in GM (Fig. 7F, G). Insignificant alteration of filamin-C in Nogo-A-silenced C2C12 cells (Fig. S5) suggests that defective myogenesis of Nogo-A-silenced cells (Fig. 6E–H) was independent with the level filamin-C and resulted from the lack of interaction with Nogo-A. Thus, in addition to its known role in cell motility via binding to F-actin and vinculin in the integrin-linked kinase (ILK) signaling pathway, our results suggest that filamin-C also plays a role in muscle differentiation via its interaction with Nogo-A (Fig. 7H, Fig. S4, and Table S6).

## Discussion

Muscles in patients with ALS show enhanced Nogo-A expression, suggesting that Nogo-A might be a disease marker^36^. However, a link between Nogo and pathophysiological conditions in skeletal muscle disorders has not yet been established.

In the present study, muscles under pathological conditions including DMD and mouse myopathy models, such as *mdx*, acute damage induced by myotoxic notexin injection, and chronic muscle damage from liver dysfunction, not only showed altered expression of myogenic factors but also consistently displayed upregulated Nogo-A and downregulated Nogo-C (Figs. 1B and 2A, E, G).

To enhance our understanding of the role of Nogo in muscle function, we analyzed normal and damaged Nogo^−/−^ muscles and found augmented inflammation under normal conditions and defective regeneration following damage in the Nogo^−/−^ muscles (Fig. 3A). Interestingly, the absence of Nogo upregulated the expression of genes involved in fat differentiation and downregulated the expression of those modulating skeletal muscle differentiation (Fig. 3B, D). Muscle regeneration is essential for maintaining muscle function after damage, and improper substitution of fat for new muscle during muscle regeneration leads to a decline in muscle integrity^37^. Muscle homeostasis is maintained via the regulation of muscle protein metabolism^38^ and might be affected by not only nutrient intake and exercise but also circadian rhythm factors^39^. Circadian rhythm is regulated via the circadian clock system located in the suprachiasmatic nucleus of anterior hypothalamus responding to environmental stimuli, including light and diet^40^. Mammals regulate the physiological functions of peripheral tissues, including skeletal muscle, via the peripheral circadian clock^41^. Surprisingly, the Nogo^−/−^ muscles showed significantly altered expression of circadian clock genes (Fig. 3F, G), but our current knowledge on the role of Nogo in circadian clock regulation is limited. Studies showing that Nogo-A-deficient transgenic rats have altered circadian activity patterns suggest a role for Nogo-A in circadian clock maintenance^42^. Therefore, dysregulated circadian clock gene expression in Nogo^−/−^ muscle may lead to defective muscle homeostasis and regeneration (Fig. 3). Previous analysis of the circadian transcriptome in mouse skeletal muscle revealed that genes essential for muscle function, including *Myod*, *Atrogin*-*1*, and *Murf-1*, were subject to circadian regulation^43^. In addition, Dyar et al.^44^ suggested the involvement of the circadian clock in energy homeostasis and lipid metabolism in skeletal muscle. CLOCK, encoded by *CLOCK* (circadian locomotor output cycles kaput), and BMAL1 (brain and muscle ARNT-like 1) are major components of the circadian machinery and regulate the expression of other clock genes including *CRY1*, *CYR2*, *PER1*, and *PER2*, after the formation of a heterodimer^40,45^. In the present study, the Nogo^−/−^ muscles exhibited downregulated levels of *Clock* and *Bmal1*, and abrogated expression of the components of the PER/CRY complex that inhibits the CLOCK-BAML1 complex, such as *Per1*, *Per2*, *Per3*, *Cry1*, and *Cry2* (Fig. 3F). In addition, the expression of *Bhlhe40*, another inhibitor of the CLOCK-BAML1 complex, was increased in the Nogo^−/−^ muscles, resulting in the upregulation in expression levels of *Cebpa* and *Cebpb*, which participate in adipogenesis. These results suggest that Nogo is critical in regulating circadian rhythm and that the absence of Nogo in muscle contributes to the enhanced expression of genes involved in fat deposition in muscle tissue (Fig. 3B, D, H).

Skeletal muscle expresses all three Nogo transcript variants, and the pathological conditions evaluated in the present study exhibited significant increases in Nogo-A and significant decreases in Nogo-C (Figs. 1B and 2A, E, G). During C2C12 myoblast differentiation, the expression levels of both Nogo-A and Nogo-C were significantly increased (Fig. 6A). Based on previous studies showing that Nogo-C acts as an apoptosis inducer in cardiac muscle following myocardial infarction^46^, damaged muscles might facilitate muscle regeneration by restricting the pro-apoptotic function of Nogo-C.

Muscle regeneration begins with the activation of satellite cells and results in myotube formation, which is mediated by serial expression of myogenic factors including Pax7, MyoD, and myogenin. Nogo-A tended to increase in parallel with other myogenic factors in myoblast myocyte differentiation into myotubes (Fig. 4A–C), and pathological conditions requiring myogenesis induced Nogo-A expression (Figs. 1B, D and 2A, C–E, G). Accordingly, we assessed if Nogo-A was a potent myogenesis regulator during muscle differentiation and found that silenced Nogo-A resulted in attenuation of C2C12 differentiation with a minimal effect on MyoD and myogenin (Fig. 6).

Therefore, the levels of predicted TFs were evaluated to determine potential regulators of Nogo-A expression, and ERRγ showed the most significant increases during myoblast differentiation (Fig. 5A, Table S4, and Fig. S2). We previously reported ERRγ as a key to myogenesis^33^ and observed that myopathic muscles increased ERRγ expression in the present study (Fig. 5B, C). This study suggests the contribution of ERRγ to Nogo-A expression in C2C12 cells by showing that silenced ERRγ downregulated Nogo-A expression (Fig. 5D).

We additionally uncovered that cytoskeleton is the predominant location of Nogo-A in myogenesis (Fig. 7B, C) and identified candidates interacting with Nogo-A during myogenesis by immunoprecipitation-mass spectrometry analysis (Table S5). Filamin-C was one of the Nogo-A interacting molecules with a known location in the cytoskeleton and its level was significantly elevated during C2C12 differentiation (Fig. 7D). Filamin-C is a muscle-specific filamin, a class of actin-binding proteins, and functions via interacting with transmembrane proteins such as δ- and γ-sarcoglycan^47^. Mutated filamin-C causes filaminopathy characterized by proximal muscle weakness due to defective myofibrils and abnormally aggregated proteins in muscle fibers^48^, and lack of filamin-C results in defective myogenesis and myotube formation^49^. The filamin-C and Nogo-A levels were increased during C2C12 differentiation, and the interaction between Nogo-A and filamin-C was also enhanced (Fig. 7F, G). Filamin-C functions via interacting with vinculin and F-actin within the ILK signaling pathway inducing cell motility (Fig. 7H and Table S6). Cytoskeletal reorganization is a pivotal process for completion of myogenesis^50^. The analyzed pathways associated with Nogo-A-interacting proteins support that Nogo-A plays a role in cellular motility and cytoskeletal organization via MYLs (Table S6 and Fig. S4). Especially, the increased interaction between Nogo-A and filamin-C during myogenesis indicates that Nogo-A involves in myogenesis by participating in cytoskeletal rearrangement (Fig. 7H). Besides, the defective myogenesis by the abrogation of Nogo-A without the alteration of the filamin-C level (Fig. S5) suggests the critical role of Nogo-A in the cytoskeletal organization during myogenesis.

In conclusion, our findings suggest that Nogo plays a pivotal role in maintaining muscle homeostasis by its involvement in the modulation of lipid metabolism, muscle cell differentiation, and circadian rhythm-related factors. Although the role of Nogo in circadian rhythm regulation remains elusive, our results suggest the novel function of Nogo in regulating muscle circadian clock genes, which might be affected by circadian rhythm determined in the suprachiasmatic nucleus. The dysregulated circadian clock appear to be contributing to abnormal muscle homeostasis in Nogo^−/−^ muscle. Furthermore, in muscles undergoing regeneration under pathological conditions, an increase in Nogo-A participates in the regenerative process by modulating myogenesis via its interaction with filamin-C.

## Materials and methods

### Patient samples

Skeletal muscle slices and tissue fragments were obtained from patients at Pusan National University Yangsan Hospital in Yangsan, Republic of Korea (Institutional Review Board No. 05-2018-045), and Yonsei University College of Medicine in Seoul, Republic of Korea (Institutional Review Board No. 3-2018-0060), after obtaining approval from the Medical School Ethics Committee. Muscle biopsies were obtained from five age-matched healthy patients as the control group (Table 1).

**Table 1:** Information on patients with myopathy and normal subjects.

#	Sex	Age (years)	Clinical diagnosis	#	Sex	Age (years)	Clinical diagnosis
D1	M	5	Inflammatory myopathy	N1	F	42	Normal
D2	M	5.6	Inflammatory myopathy	N2	F	26	Normal
D3	M	1.83	DMD (exon 45 deletion)	N3	F	41	Normal
D4	M	5.1	Inflammatory myopathy	N4	F	18	Normal
D5	M	2	Dysferlinopathy	N5	F	1.5	Normal
D6	F	81	Inflammatory myopathy				
D7	F	46	Inflammatory myopathy				
D8	F	57	Inflammatory myopathy				
D9	M	15	DMD (exon 45–52 deletion)				
D10	M	20	DMD (exon 50 deletion)				

### Animal models

All mice were housed under appropriate conditions with a 12 h light/12 h dark cycle and access to water ad libitum in accordance with Kyungpook National University animal facility regulations. For experiments modeling DMD, 12-week-old male WT (C57BL/10J) and *mdx* mice (C57BL/10ScSn-Dmdmdx/J) were utilized. The *mdx* mice were kindly provided by Jacques P. Tremblay (CHUQ Research Center, Quebec City, Canada) and C57BL/10SnSlc mice were purchased from Japan SLC (Hamamatsu, Japan), as previously described^51^. Nogo^−/−^ mice, previously described^52^, were generously provided by Binhai Zheng (University of California, San Diego, CA, USA). Animal experiments and protocols above were approved by the Institutional Animal Use and Care Committee of Kyungpook National University in Daegu, Republic of Korea (KNU 2014-0167, KNU 2018-0105), and conformed to the policies and guidelines of the National Institutes of Health (NIH). The intragastric (iG) ethanol feeding model was provided by the Animal Core Tissue Sharing Program at the NIAAA-funded Southern California Research Center for ALPD and Cirrhosis (P50AA011999).

### Mouse model of muscle abnormality

#### Notexin-induced muscle injury model

For notexin-induced muscle injury, 8-week-old male WT (C57BL/6 J, Nogo^+/+^, *n* = 5) and Nogo KO (Nogo^−/−^, *n* = 5) mice were intramuscularly injected with notexin (500 ng/mL in phosphate-buffered saline (PBS); Latoxan) as a single dose of 50 μL into the gastrocnemius muscle. Mice were killed at 8 days post injection and gastrocnemius muscles were collected as previously described^53^.

#### ALD mouse model

The previously described mouse model of ALD (HCFD + Alc + Binge)^54^ includes 2 weeks of feeding with a Western diet to produce peak fibrogenic response in liver, 1 week for iG surgery and recovery, and 8 weeks on weekly HCFD/alcohol hybrid binges for a total of 11 weeks.

#### Cell culture and myogenic differentiation

C2C12 myoblast cells, an immortalized mouse myoblast cell line, were cultured in Dulbecco’s modified Eagle’s medium (DMEM, Corning, #10-013-CUR) supplemented with 10% fetal bovine serum (Corning, #35-015-CU) and 100 U/mL penicillin–streptomycin (Gibo-BRL, #15140122) in a humidified atmosphere containing 5% CO_2_ at 37 °C. To induce myotube differentiation, cells were washed with PBS and cultured with DM (DMEM containing 2% horse serum [Gibco-BRL, #16050-122] and 100 U/mL penicillin–streptomycin). iMSCs were developed from mouse embryonic fibroblasts and sort-iMSCs were obtained by sorting iMSCs with stem cell markers including CD106 (Pharmingen, #01812D) and α7 integrin (Santa Cruz, sc-50431), as previously described^33^.

### Preparation and staining of frozen muscle tissue sections and cells

#### Preparation of muscle tissue sections

Muscle tissues were fixed in 4% paraformaldehyde (Sigma) in PBS for 24 h and immersed in gradient sucrose solutions (30% and 35%) in PBS for 6 and 16 h, respectively. To prepare muscle cryoblocks, tissues were blocked in OCT compound (Leica, #3801480), stored at −70 °C, sectioned into 4–5 μm slices with a Leica cryostat, mounted on slides, and dried under room air for 30 min. Prepared sections were kept at −70 °C until staining. Muscle paraffin blocks were prepared following dehydration of the tissues with an ethanol series, embedding in paraffin, and sectioning at 4 μm-thick slices with a Leica rotary microtome.

#### Hematoxylin/eosin staining of muscle sections

Histopathologic evaluation was performed on sections stained by routine hematoxylin/eosin staining. Cryosectioned muscle tissues were rehydrated with PBS and paraffin-embedded sections were rehydrated by immersing in ethanol. After rinsing with H_2_O, tissues were stained in hematoxylin solution for 5 min, washed with H_2_O, and counter-stained with eosin. Mounting was followed by dehydration with xylene and images were captured in digital format using a Leica DM5000B microscope.

#### Immunofluorescence staining of muscle sections

Prepared sections were rehydrated in PBS, boiled in 10 mM citric acid buffer (pH 6.0) at 95 °C for 10 min to unmask antigens, cooled down at room temperature for 30 min, washed with PBS, and permeabilized with 0.1% Triton-X (Sigma-Aldrich, #9002-93-1) in PBS for 5 min. Next, the sections were blocked with 5% donkey serum in PBS for 1 h and incubated with a cocktail primary antibodies in blocking buffer for overnight at 4 °C. After washing with PBS, a cocktail of fluorescence-conjugated secondary antibodies (Goat anti-rabbit-IgG Alexa Flour555-conjugated, Abcam, ab150078; Goat anti-mouse-IgG Alexa Flour488-conjugated, Abcam, an150114; Goat anti-mouse-IgG Alexa Fluor594-conjugated, Thermo Fisher, A-11005; Goat anti-rabbit-IgG FITC-conjugated, Abcam, ab6717) were allowed to bind for 1 h at room temperature. After washing with PBS, the sections were mounted using antifade mounting solution with DAPI (Cell Signaling, #8961) and images were captured using a confocal laser scanning microscope (LSM700; Carl Zeiss).

#### Immunofluorescence staining of cells

After the removal of culture medium, cells were washed with PBS and fixed with 4% paraformaldehyde for 10 min. The cells were permeabilized by incubation in ice-cold methanol for 10 min and were washed with PBS. Next, the cells were blocked using 5% donkey serum in PBS, incubated in a cocktail of primary antibodies diluted in blocking solution overnight at 4 °C, and incubated in a cocktail of fluorescence-conjugated secondary antibodies for 1 h. Coverslips were mounted with an antifade mounting solution containing DAPI and images were captured with a confocal laser scanning microscope.

#### siRNA transfection

Gene silencing was achieved by transfection of specific siRNAs against Nogo-A and ERRγ in C2C12 myoblast cells. siRNAs for mouse Nogo-A (si-Nogo-A) and mouse ERRγ (si-ERR) were chemically synthesized (Bioneer, Table 2), and premade negative control siRNA (si-scrambled, Bioneer, SN-1011) were purchased. C2C12 cells were plated on six-well plates at 1 × 10^5^ cells per well, cultured to 40% confluency, and incubated with culture medium without antibiotics for 2 h before transfection. Lipofectamine 2000^®^ reagent (1 : 100, Invitrogen, #11668027) and siRNAs (50 nM) were diluted in Opti-MEM (Thermo Fisher Scientific, #31985062) according to the manufacturer’s instructions and the mixture was added to culture medium for 6 h. After removing the siRNA mixture from the culture medium, the cells were cultured with GM for 2 days. Next, the medium was changed to DM to induce myoblast differentiation over 3 days.

**Table 2:** Sequences of siRNA.

Mouse siRNA	Sequence (5′→3′)
Sense siRNA-ERRγ	CCU CUG AUU GUA UGG AAC AUU UCU U
Antisense siRNA-ERRγ	AAG AAA UGU UCC AUA CAA UCA GAG G
Sense siRNA Nogo-A	CAA AGA GGA UUU AGU UUG UAG UGC A
Antisense siRNA Nogo-A	UGC ACU ACA AAC UAA AUC CUC UUU G

### RNA isolation and quantitative PCR analysis

#### RNA isolation

Total RNA was isolated from cells and muscle tissues of mice and human samples using TRIzol^®^ reagent (Invitrogen), according to the manufacturer’s instructions.

#### Quantitative RT-PCR

cDNA was synthesized from 2 µg total RNA using Maxima First Strand cDNA synthesis kit for RT-qPCR (Thermo Fisher Scientific), according to the manufacturer’s instructions. The gene expression levels were analyzed by quantitative reverse-transcription PCR using SYBR Green (TOPreal qPCR premix, Enzynomics) and a Rotor-Gene Q instrument (Qiagen) or Bio-Rad CFX Connect Real-Time System (Bio-Rad). The expression levels of gene transcripts were normalized to *GAPDH* (human) or *18S rRNA* (mouse), and the results were evaluated by the Rotor-Gene Q series software (Qiagen) or Bio-rad CFX Maestro (Bio-Rad).

### Protein extraction and protein detection

#### Muscle protein extraction

Muscles from humans and mice were lysed with lysis buffer (50 mM Tris-HCl pH 7.4, 150 mM NaCl, 1 mM EDTA, 5 mM Na_3_VO_4_, 20 mM NaF, 10 mM sodium pyrophosphate, protease inhibitor cocktail [Roche, #04693132001], 1 mM phenylmethylsulfonyl fluoride (PMSF) [Generay Biotech, #0754-PMSF], 1% Triton-X, and 0.1% SDS [Biosesang, #S2003]) and homogenized using a homogenizer. After centrifugation (15,000 × *g* for 15 min at 4 °C) to remove debris, total protein concentration of the supernatants was determined with the Pierce BCA protein assay kit (Thermo Scientific, #K1672).

#### Cell protein extraction for western blotting

Cells in 6-well plates were rinsed with PBS and lysed with 200 µL cell lysis buffer (1× RIPA buffer [Cell Signaling, #9806], 1 mM PMSF, and protease inhibitor cocktail). The lysates were incubated at 4 °C with rocking for 30 min to facilitate protein extraction. After centrifugation (15,000 × *g* for 15 min at 4 °C) to remove debris, total protein concentration of the supernatants was determined with the BCA protein assay kit.

#### Western blotting

Protein concentrations of the samples were adjusted to 2–3 µg/µL, which were mixed with an appropriate volume of 5× SDS loading buffer, boiled, and electrophoresed on SDS polyacrylamide gels (10% or 12%). The separated proteins were transferred to nitrocellulose membranes (0.2 µm pore size; Amersham), stained with ponceau solution (Translab, TLP-113), washed with H_2_O, and incubated in blocking buffer (1% bovine serumalbumin in Tris-buffered saline containing 0.1% Tween 20) for 1 h. The membranes were incubated with primary antibodies (anti-Nogo-A, Abcam, #ab62024; anti-MyoD, Santa Cruz, #sc-32758; anti-Myogenin, Santa Cruz, #sc-12732; anti-Pax7, Santa Cruz, #81975; anti-S1PR2, Proteintech, #21180-a-AP) diluted to 1 : 1000 in blocking buffer overnight at 4 °C, followed by incubation with appropriate secondary horseradish peroxidase-conjugated antibodies for 1.5 h. The protein levels were determined by visualization with ECL reagent (Thermo Fisher Scientific, #34076). The bands were imaged with a chemiluminescence imager (Amersham) and band intensities were analyzed using the NIH ImageJ software.

#### Enzyme-linked immunosorbent assay

Isolated muscles were transferred to the ProEX CETi lysis buffer (Translab, TLP-121CETi) and homogenized with a homogenizer, and the supernatants were collected after centrifugation at 15,000 × *g* for 15 min at 4 °C. The amount of IL-6 was measured using a Mouse IL-6 Platinum ELISA kit (Invitrogen, KMC0061), according to the manufacturer’s instructions.

#### Immunoprecipitation with Nogo-A or S1PR2 antibody

The lysed cells in lysis buffer (500 µL/100 mm culture dish; 15 mM HEPES, 145 mM NaCl, 10 mM EDTA, 0.1 mM MgCl_2_, 2 mM Na_3_VO_4_, 2% Triton-X, protease inhibitor cocktail, 1 mM PMSF). The cell lysates (1000 µg total protein) were precleared with protein A-coated agarose beads (Cell Signaling, #9863) or protein G-coated Dynabeads^®^ (Invitrogen, #10003D) for 1 h at 4 °C and immunoprecipitated with 5 µg Nogo-A antibody (Abcam, #ab62024) or 2 µg of S1PR2 antibody (Proteintech, #21180-a-AP) overnight at 4 °C with rotation. Immune complexes were collected by incubation with agarose beads Dynabeads for 3 h at 4 °C with rotation, respectively. The collected beads were washed with lysis buffer and boiled after adding 1× SDS loading buffer. Western blotting was performed to detect Nogo-A, filamin-C, and S1PR2 with control lysates containing 100 µg total protein and 30 µL immunoprecipitated samples under reducing conditions.

### RNA sequencing and network analysis

#### mRNA sequencing and data analysis

Total RNA was isolated from the gastrocnemius muscle tissues of Nogo^+/+^ and Nogo^−/−^ mice (three biological replicates per group) using the RNeasy mini kit (Qiagen). mRNA was isolated from total RNA and fragmented using the Illumina Truseq™ Stranded mRNA LT sample prep kit, and reverse transcription of the mRNA fragments was performed using Superscript III reverse transcriptase (Life Technologies). Adaptor-ligated libraries were generated according to the manufacturer’s protocol and sequenced using a HiSeq 2500 system. To preprocess read sequence data, adaptor sequences (TruSeq universal and indexed adapters) were trimmed by *cutadapt*. The trimmed data were aligned to the reference mouse genome (GRCm38.p6) using the STAR aligner^55^ and quantified to the gene features (GRCm38.95.gtf) in fragments per kilobase of transcript per million fragments of mapped values (FPKM) using the Cufflinks software.

#### Identification of DEGs

An expressed gene was defined as that with an FPKM ≥ 1 for at least one of the six samples. For these expressed genes, log_2_(FPKM) values were normalized using the quantile normalization method^56^. To identify DEGs among the expressed genes, their combined adjusted *p*-values were calculated as previously described^57^. In brief, for each gene, we first calculated the *t*-statistic using Student’s *t*-test and the log_2_-median ratio test (log_2_ fold change) by comparing log_2_(FPKM) values between the Nogo^+/+^ and Nogo^−/−^ samples. We then generated empirical KO distributions of *t*-statistic values and log_2_ fold changes by randomly permuting the 6 samples 1000 times. Based on two empirical KO distributions for each gene, we computed adjusted *p*-values for the Student’s *t*-test and the median ratio test, and combined these adjusted *p*-values using Stouffer’s method. Genes with combined adjusted *p*-values < 0.05 and log_2_ fold changes larger than the cutoff (absolute mean of 2.5th and 97.5th percentile values from the empirical distribution of log_2_ fold changes; 1.3 fold change) were selected as DEGs.

#### Functional enrichment analysis

To identify cellular processes and pathways represented by the selected DEGs, we performed enrichment analysis of GOBPs and GOMFs, and determined KEGG pathways using the DAVID software. The GO terms and KEGG pathways with *p*-values < 0.05 were defined as being enriched by the DEGs.

#### Identification of key TFs

To identify major TFs responsible for the DEGs, we first collected TF-target information from TRED, Amadeus, HTRIdb, MSigDB, EEDB, bZIPDB, and MetaCore™ (GeneGo, St. Joseph, MI, USA). Human TF-target relationships were converted into mouse TF-target relationships using MGI human-mouse homolog mapping. Using these TF-target relationships, we finally selected major TFs that regulated a significant (*p* < 0.05) number of DEGs (at least five) based on Fisher’s exact test.

#### Network analysis

To build a network model describing the effect of Nogo on muscular dystrophy or cell differentiation, we first selected DEGs involved in the GOBPs and GOMFs, as well as the KEGG pathways related to these processes. We then collected the data on protein–protein interactions for these selected DEGs from the following protein–protein interactome databases: BioGrid, ConcensusPathDB, DIP, HitPredict, IntAct, MINT, and STRING. Using the information on the protein–protein interactions for the selected DEGs, we constructed a network model using *Cytoscape* and arranged the nodes according to the pathway information from the KEGG pathway database.

### Immunoprecipitation-mass spectrometry analysis

#### Sample preparation and analysis with liquid chromatography linked to tandem mass spectrometry

Immunoprecipitation with Nogo-A was conducted using MS-Compatible Magnetic IP Kit (Thermo Scientific, #90409). Protein concentrations of immunoprecipitated samples were determined by the BCA protein assay kit. Proteins were reduced with 10 mM DL-Dithiothreitol at 56 °C and alkylated with 50 mM iodoacetamide (Sigma-Aldrich, I1149) in the dark at room temperature. Next, the proteins were diluted to 100 mM with ammonium bicarbonate (Sigma, A6141), followed by the addition of trypsin (Promega) at an enzyme:protein ratio of 1:50 and incubation overnight at 37 °C. After digestion, the samples were acidified with 10% trifluoroacetic acid (TFA, Pierce, #28904), and desalted on a tC18 plate. The tC18 plate was prepared by activation with 0.1% TFA and 80% acetonitrile (Macron, Avantor Performance Materials, MK-H076-10), and equilibration with 0.1% TFA. Tryptic peptides were loaded on the plate and washed three times with 0.1% TFA. The peptides were eluted with 0.1% TFA and 80% acetonitrile (ACN), and lyophilized by vacuum centrifugation.

Approximately 1 µg of the peptides were loaded and captured on EASY-Spray^TM^ LC column (PepMapTM RSLC C18, 2 µm, 100 Å, 50 µm × 15 cm) and gradient-eluted into a Q-Exactive PLUS mass spectrometer (Thermo Scientific) using a 40 min organic gradient (5–40% B, *A* = 0.1% formic acid (FA), *B* = ACN with 0.1% FA). The electrospray voltage was set at 2.2 kV. The Q-Exactive PLUS was programmed to operate in a data-dependent mode such that the 20 most abundant precursors in each MS scan (AGC target, 3E6; maximum fill time, 100 ms; and resolution, 70 K) were subjected to higher-energy collisional dissociation (HCD) (isolation, 1.7 Da; normalized collision energy, 27%; resolution, 7.5 K; AGC target, 1E5; maximum injection, 50 ms, and intensity threshold, 1.6E5). Dynamic exclusion was enabled with a repeat count of 1 and exclusion duration of 30 s.

#### Processing of mass spectrometry data

Native RAW data files from the Q-Exactive PLUS mass spectrometer were processed using Proteome Discoverer v3.0. All data were searched against a forward-reverse mouse database assembled from the SWISSPROT database. For de-isotoped HCD spectra, the precursor mass tolerance was set to 10 ppm and the MS/MS fragment ion tolerance was set to 0.02 Da. Search parameters included trypsin specificity, with a maximum of two missed cleavages, fixed carbamidomethylation of Cys (C, + 57 Da), and variable oxidation on Met (M, + 16 Da). Reported peptide sequences were filtered based on a 1% false discovery rate.

The proteins identified to interact with Nogo-A were analyzed with the ingenuity pathways analysis program (Qiagen, https://www.qiagenbioinformatics.com/products/ingenuity-pathway-analysis), to evaluate pathways in which the differentiation state-specific Nogo-A-interacting proteins were involved. Proteins in the proteomic data were mapped to corresponding gene objects in the ingenuity pathways knowledge base. Scores were derived from the *p*-value of the test and indicated the likelihood of the mapped genes in a network being found together due to random chance (score, −log_10_p).

### Prediction of TFs regulating Nogo expression

To predict TFs that potentially regulate Nogo expression, the TF-binding site sequence of Nogo (ENSMUSR00000513840) was obtained from the Ensembl database and analyzed via the JASPAR database. Predicted factors were listed based on a relative score of >0.9.

### Statistical analysis

Statistical differences between control and experimental samples were evaluated using unpaired Student’s *t*-test (two-tailed). All results were expressed as means ± SEM. *P*-values < 0.05 were considered to indicate statistical significance.

## Supplementary information

Supplemental material legends

Figure S1

Figure S2

Figure S3

Figure S4

Figure S5

Figure S6. Graphical Abstract

Table S1

Table S2

Table S3

Table S4

Table S5

Table S6
