# Supplementary material for: Nogo-A regulates myogenesis via interacting with Filamin-C
Source: Cell Death Discov. 2021 Jan 6;7:1. doi: 10.1038/s41420-020-00384-x (PMC7791112; doi:10.1038/s41420-020-00384-x)
Supplement: Supplementary file 1 — Supplemental material legends [file 41420_2020_384_MOESM1_ESM.docx]

**Supplemental Materials**

**Figure S1. Related to Figure 2. Development of the mouse model for ALD by feeding with a diet of HCFD and alcohol**

**(A)** Hepatomegaly was assessed by measuring the ratio of liver weight (LW) to body weight (BW) (control, n = 6; ALD, n = 9). Mean ± SEM. ****p* < 0.001. **(B)** Hepatic macro- and microvesicular steatosis (control, n = 6; ALD, n = 9). Mean ± SEM. ****p* < 0.001. **(C)** Plasma levels of ALT and AST (control, n = 6; ALD, n = 9). Mean ± SEM. ***p* < 0.05.

**Figure S2. Related to Figure 5. The expression of predicted TFs involved in Nogo-A expression**

qRT-PCR analysis of specific TFs in differentiated C2C12 cells. Mean ± SEM. **p* < 0.05, ***p* < 0.01, ****p* < 0.001.

**Figure S3. Related to Figure 7. Absence of the interaction between Nogo-A and S1PR2 in C2C12 cells**

Immunoprecipitation with S1PR2-specific antibody was performed using C2C12 cell lysates in a pre-differentiated (cultured in GM) or differentiated (cultured in DM for three days) state. Input, total lysate control; IP, S1PR2 antibody-bound sample. WB analysis of the immunoprecipitated samples for indicated proteins.

**Figure S4. Related to Figure 7 and Table S5. ILK signaling pathway of muscle function involving Nogo-A-binding proteins**

**Figure S5. Insignificant effect of Nogo-A-silencing on the level of Filamin-C during differentiation of C2C12 cells**

Expression of Nogo-A in C2C12 myoblast was silenced using si-Nogo-A, and myoblast differentiation was induced by DM for three days. The level of Nogo-A and Filamin-C in differentiated Nogo-A-silenced cells by WB analysis (n = 2/group).

**Figure S6. Graphical Abstract.**

Nogo deficiency results in a dystrophic muscle phenotype and the dysregulation of muscle circadian clock components, including BMAL1, NPAS2, and CLOCK, implicating a role for Nogo in myogenesis and lipid storage. Myoblasts expressing Nogo-A undergo a well-organized myotube differentiation process in the presence of an interaction between Nogo-A and filamin-C. Conversely, Nogo-deficient myoblasts exhibit defective myogenesis, which results in impaired muscle homeostasis including dysregulated myogenesis and increased fat deposition.

**Table S1. Related to Figure 3. DEGs between Nogo^−/−^ and Nogo^+/+^ mice**

A total of 703 DEGs were altered in the Nogo^−/−^ mice compared to the Nogo^+/+^ mice. Ensembl IDs, Entrez IDs, Gene symbols, muscle markers, and FPKM values for each sample are shown. For each group, the DEG columns show which genes are DEGs (1, upregulated; −1, downregulated). FC columns show log_2_ fold changes between the Nogo^−/−^ and Nogo^+/+^ mice.

**Table S2. Related to Figure 3. Results of functional enrichment analysis by DAVID**

For each term from the KEGG pathway, GOBP, and GOMF in up- and down-regulated genes, total number of genes, *p* values, and gene lists are shown.

**Table S3. Related to Figure 3. Results of TF enrichment analysis**

For each TF, number of TFs targeting DEGs, *p* values based on Fisher’s exact test, and gene lists are shown.

**Table S4. Related to Figure 5. Prediction of TFs involved in Nogo-A expression**

TF binding sites on *Nogo-A* were analyzed using the JASPAR database (http://jaspar.genereg.net/), and TFs with a relative score greater than 0.9 were selected.

**Table S5. Related to Figure 7. Nogo-A-binding proteins during C2C12 myoblast differentiation**

Nogo-A-binding proteins in proliferating or differentiated C2C12 cells were analyzed by immunoprecipitation-mass spectrometry with a Nogo-A-specific antibody. List shows differentiated cell-specific Nogo-A binding proteins.

**Table S6. Related to Figure 7. Signaling pathway related to Nogo-A-binding proteins**

Signaling pathway implicated in muscle homeostasis in which Nogo-A-binding proteins (Table S5) are involved.
