# Supplementary figures and images for: Nogo-A regulates myogenesis via interacting with Filamin-C
Source: Cell Death Discov. 2021 Jan 6;7:1. doi: 10.1038/s41420-020-00384-x (PMC7791112; doi:10.1038/s41420-020-00384-x)

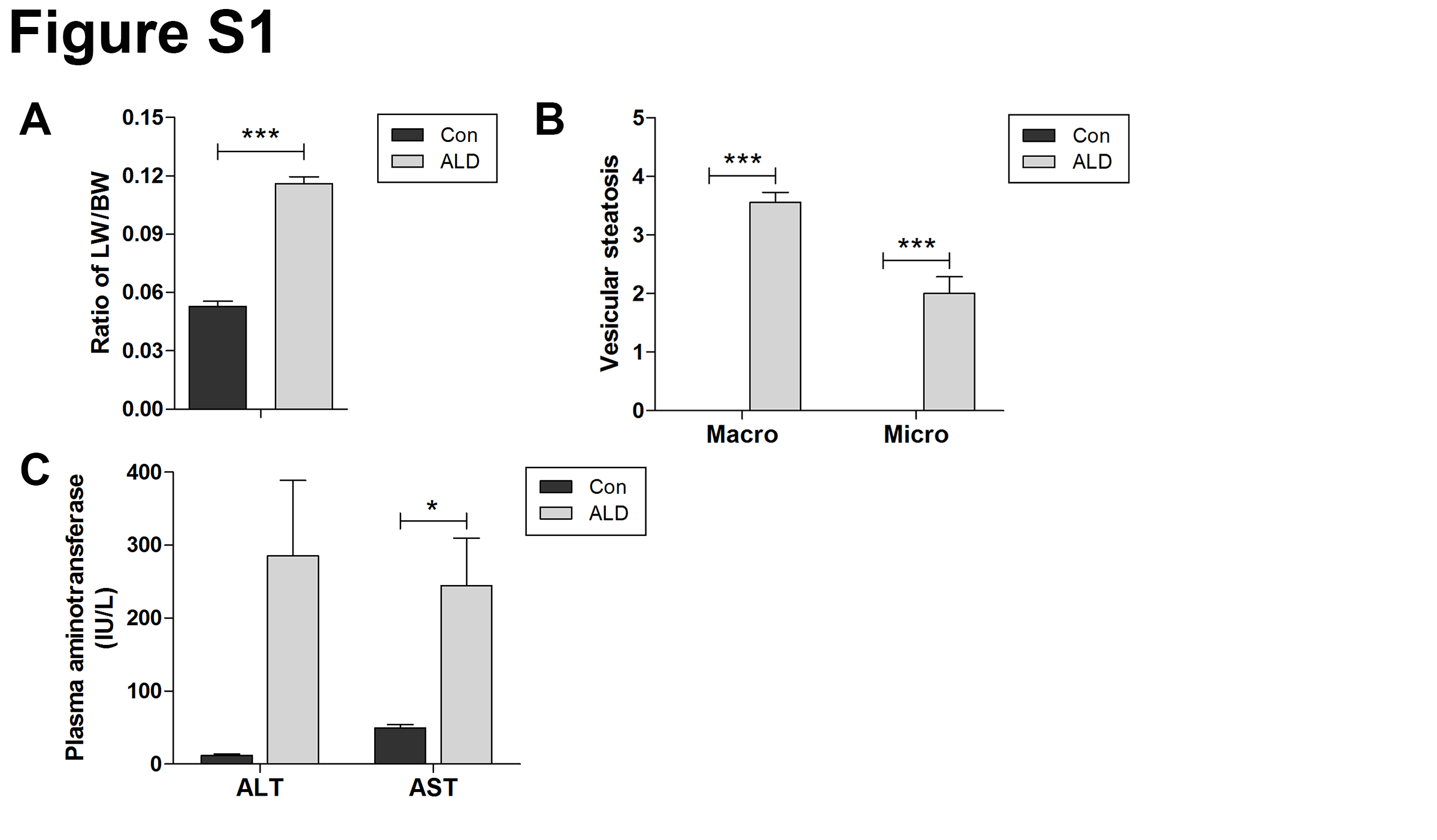

Supplement: Supplementary file 2 — Figure S1 [file 41420_2020_384_MOESM2_ESM.tif]

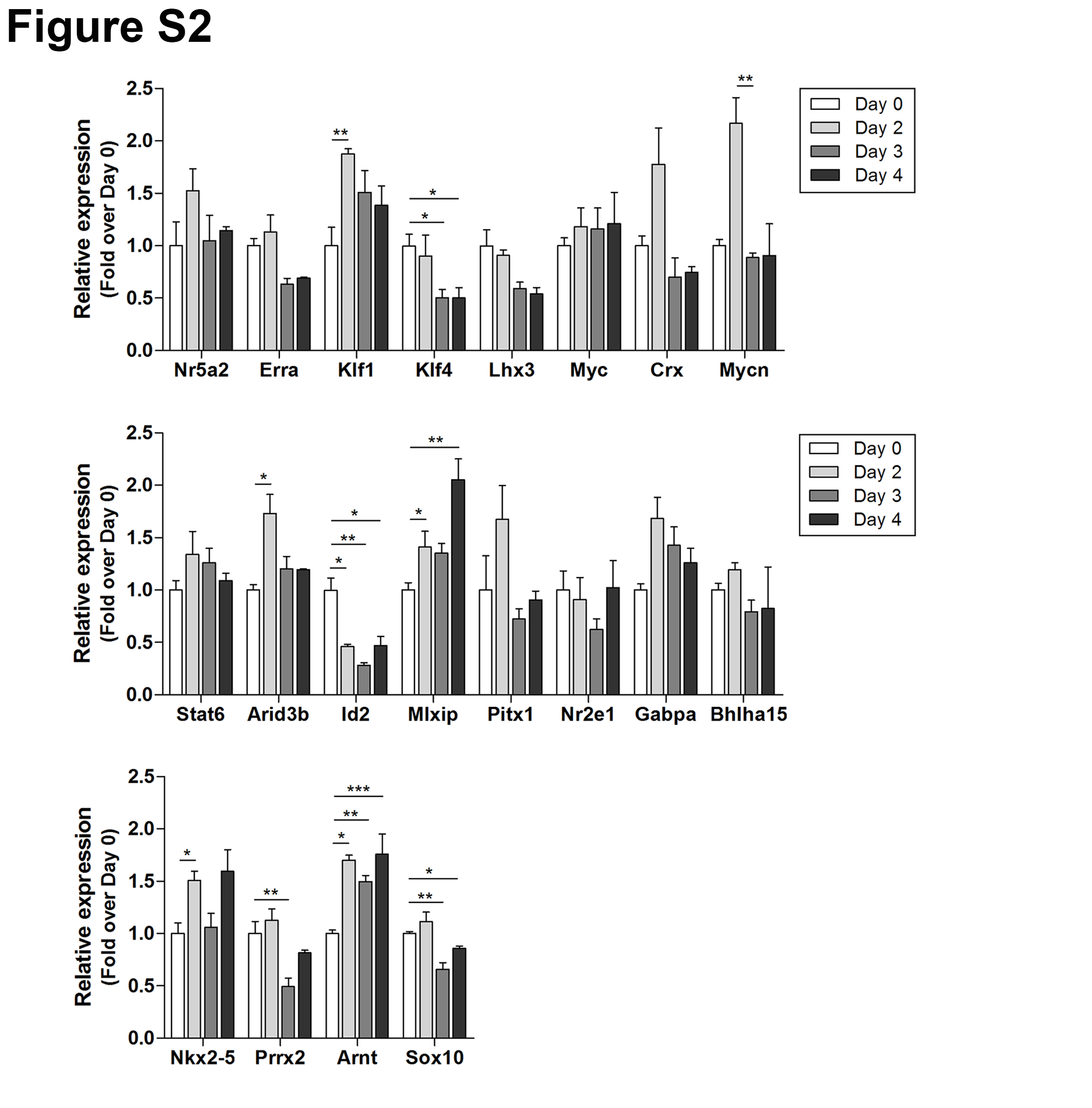

Supplement: Supplementary file 3 — Figure S2 [file 41420_2020_384_MOESM3_ESM.tif]

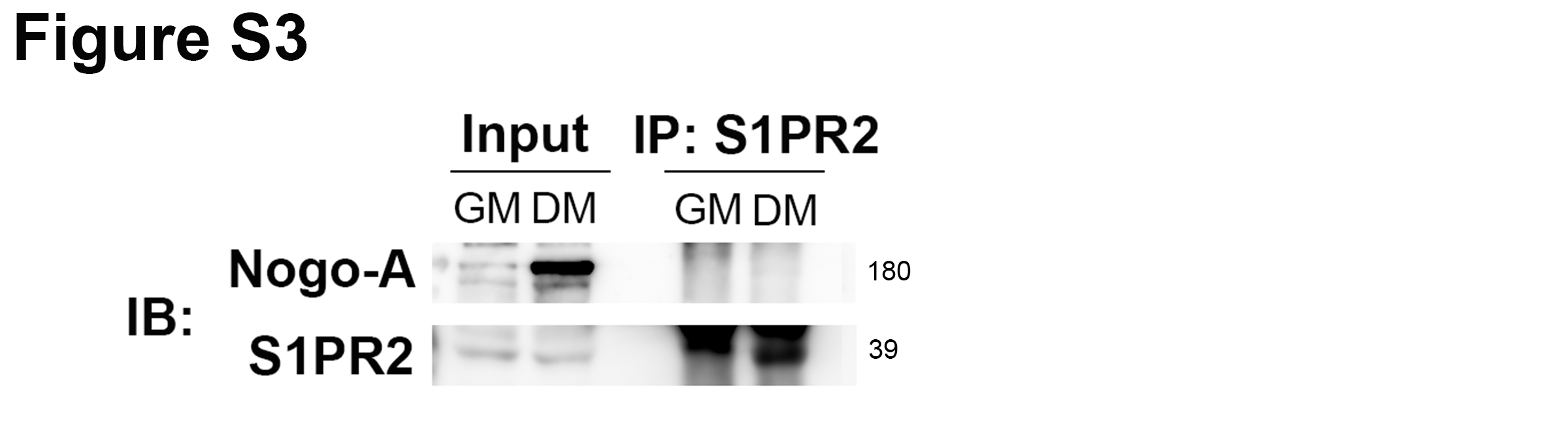

Supplement: Supplementary file 4 — Figure S3 [file 41420_2020_384_MOESM4_ESM.tif]

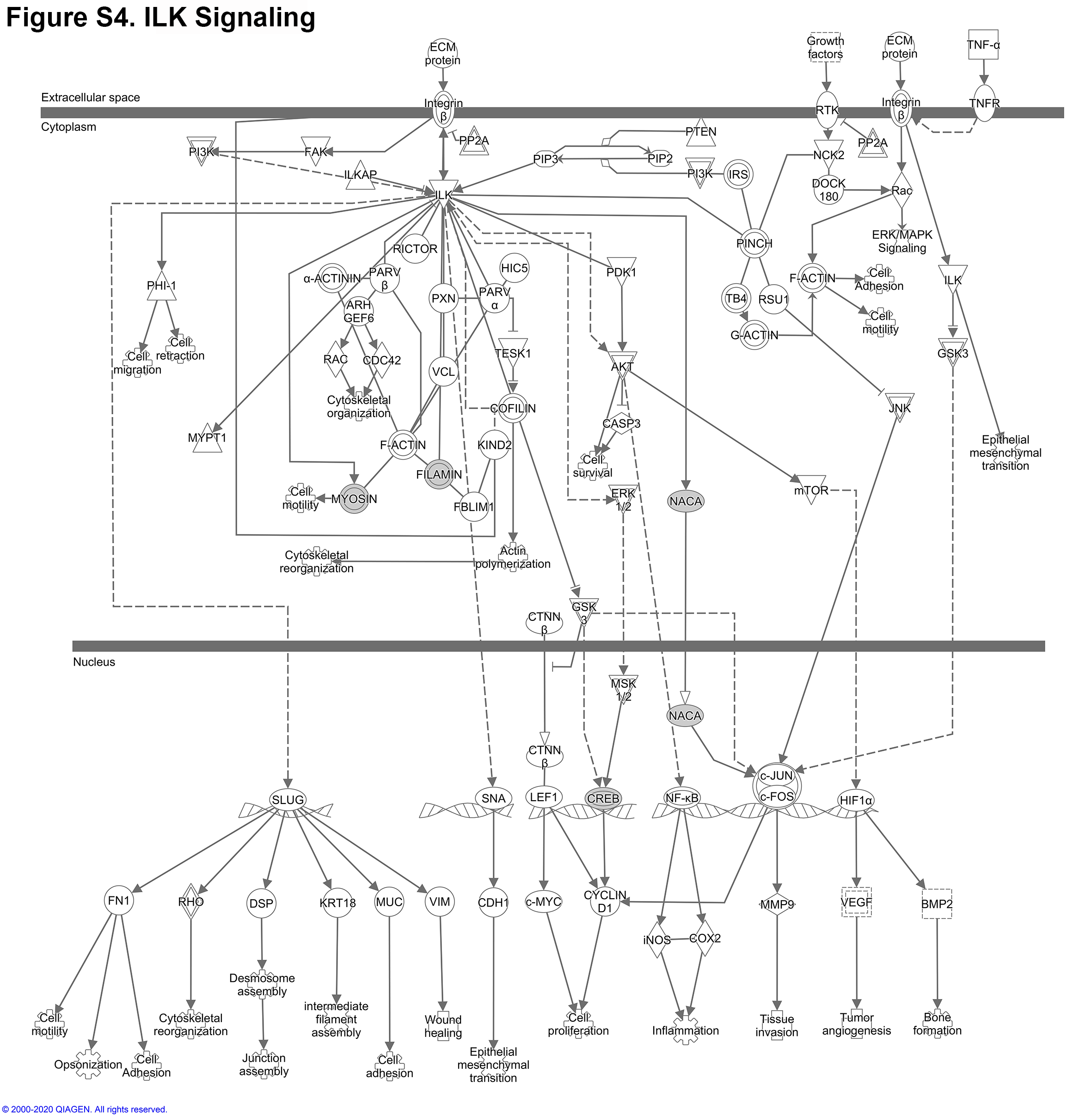

Supplement: Supplementary file 5 — Figure S4 [file 41420_2020_384_MOESM5_ESM.tif]

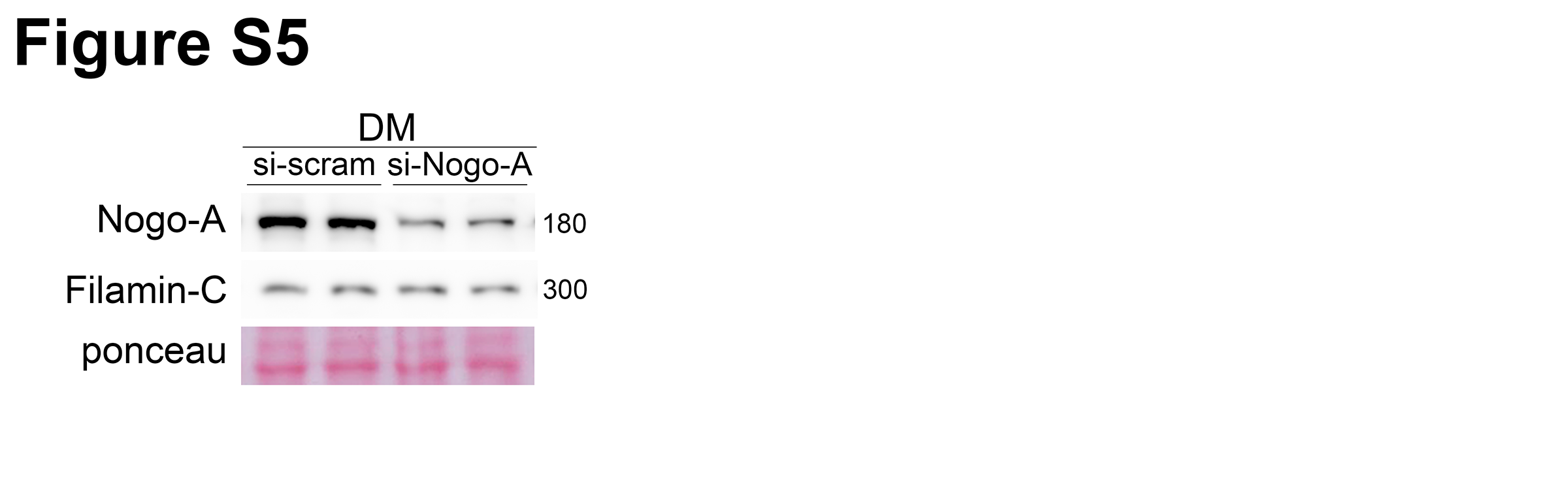

Supplement: Supplementary file 6 — Figure S5 [file 41420_2020_384_MOESM6_ESM.tif]

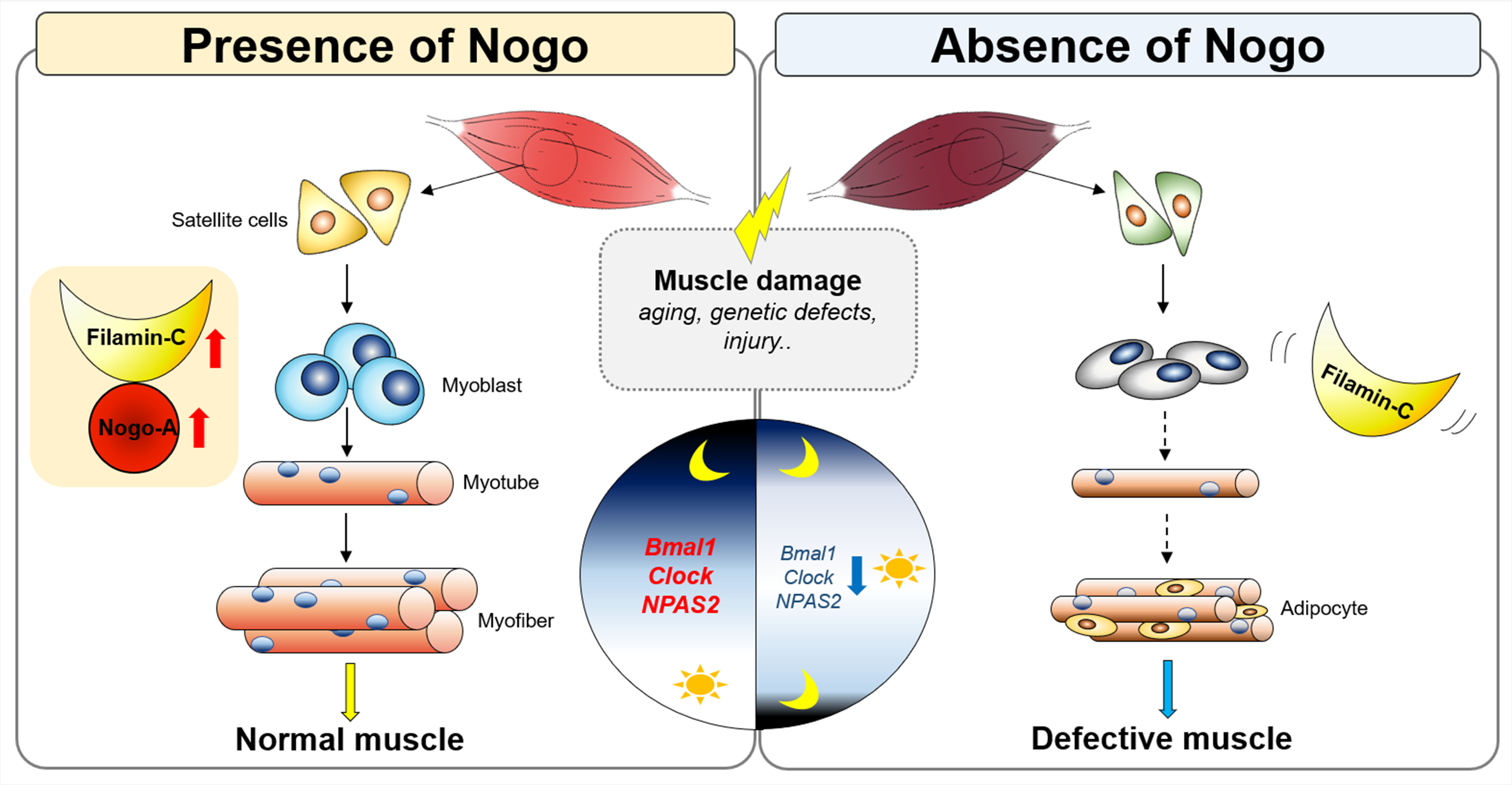

Supplement: Supplementary file 7 — Figure S6. Graphical Abstract [file 41420_2020_384_MOESM7_ESM.tif]

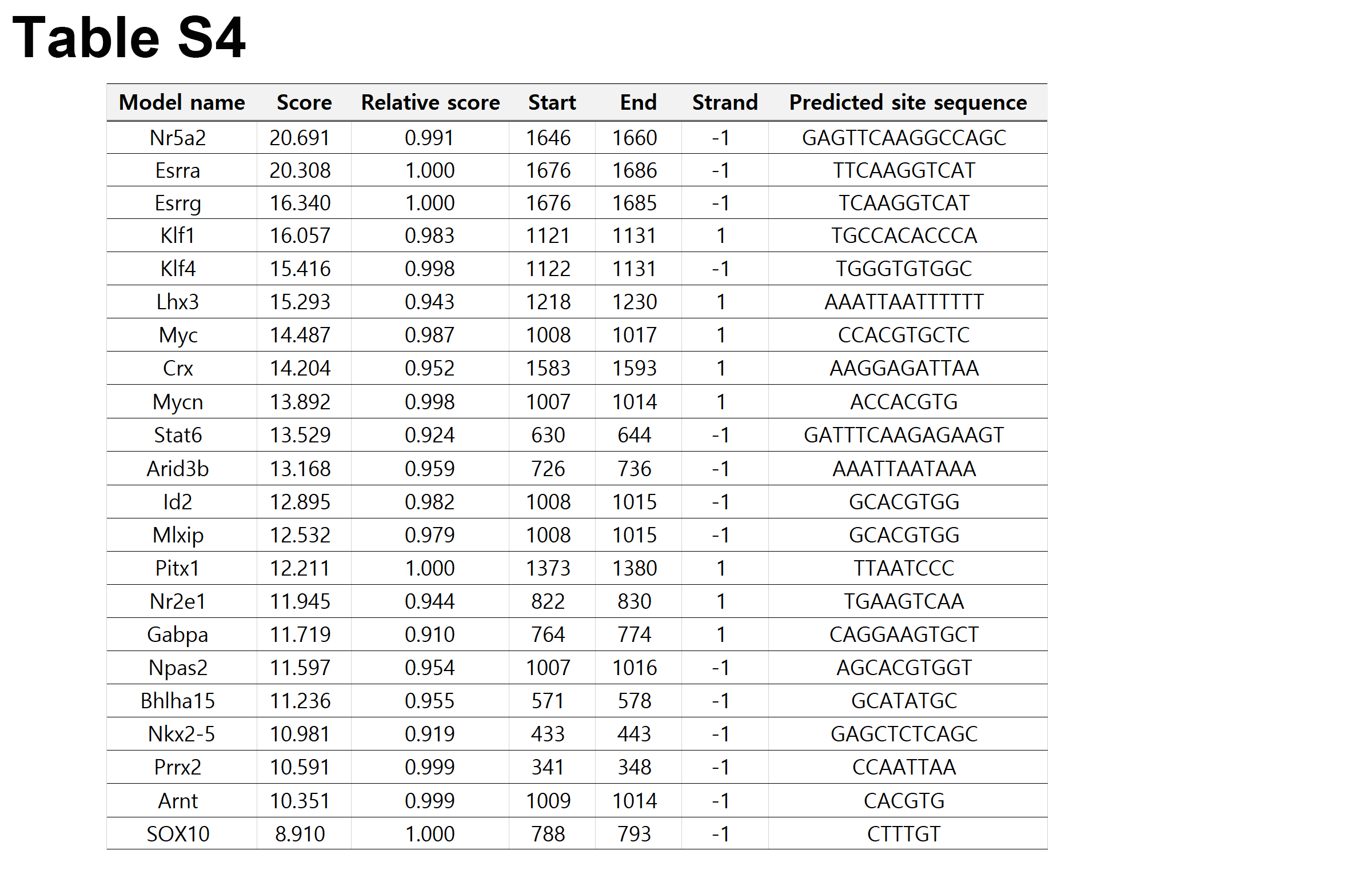

Supplement: Supplementary file 11 — Table S4 [file 41420_2020_384_MOESM11_ESM.tif]

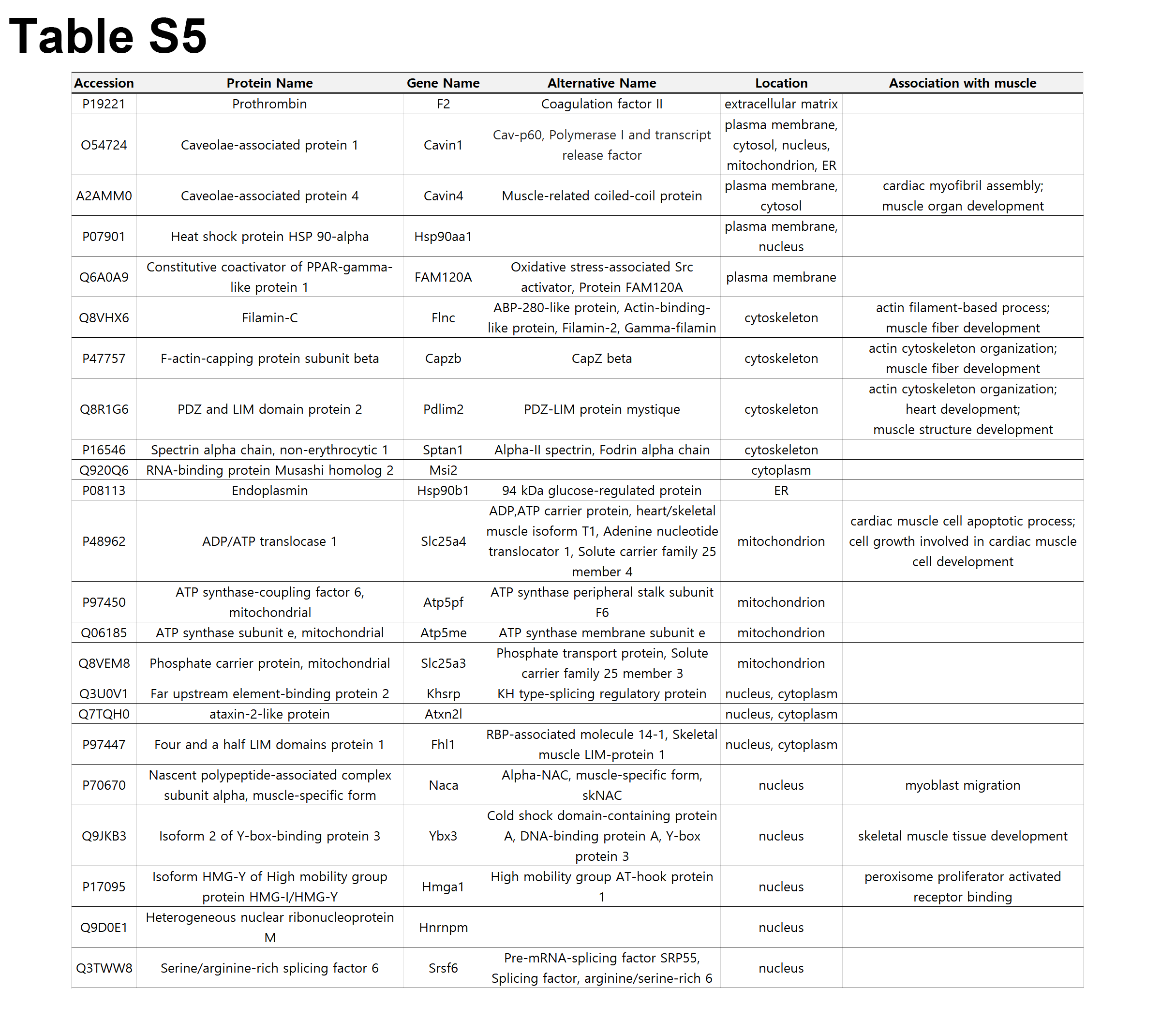

Supplement: Supplementary file 12 — Table S5 [file 41420_2020_384_MOESM12_ESM.tif]

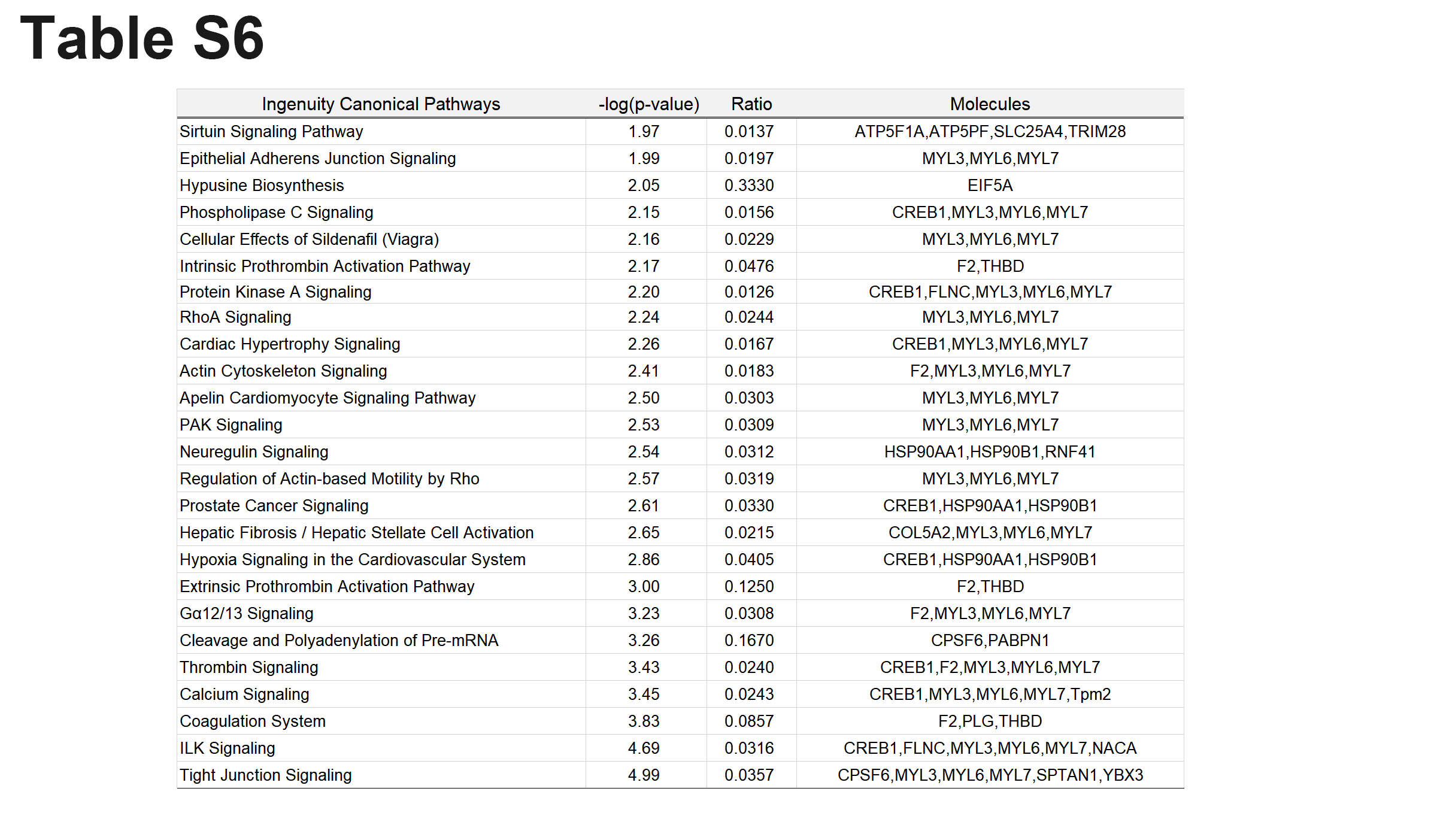

Supplement: Supplementary file 13 — Table S6 [file 41420_2020_384_MOESM13_ESM.tif]
